# *In vivo* emergence of ceftazidime/avibactam resistance in *Pseudomonas aeruginosa* driven by OXA-1381, a novel W157L Ω-loop variant of OXA-2

**DOI:** 10.1128/aac.00584-26

**Published:** 2026-08-19

**Authors:** Gloria Pérez-Rodríguez, Lucía González-Pinto, Pablo Aja-Macaya, Carlos Molina-Cáceres, Lucía Sánchez-Peña, Tania Blanco-Martín, Antonio Oliver, Germán Bou, Jorge Arca-Suárez

**Affiliations:** 1 Servicio de Microbiología Clínica & Grupo de Investigación en Microbiología, Instituto de Investigación Biomédica de A Coruña (INIBIC), Complexo Hospitalario Universitario de A Coruña (CHUAC), SERGAS, Universidade da Coruña (UDC) 16737, A Coruña, Spain; 2 CIBER de Enfermedades Infecciosas (CIBERINFEC), Instituto de Salud Carlos III 38176, Madrid, Spain; 3 Servicio de Microbiología, Servicio de Microbiología and Unidad de Investigación, Hospital Universitario Son Espases, Instituto de Investigación Sanitaria Illes Balears (IdISBa) 375118, Palma de Mallorca, Spain; 4 Departamento de Fisioterapia, Medicina y Ciencias Biomédicas, Universidade da Coruña 16737, A Coruña, Spain; University of Fribourg, Fribourg , Switzerland

**Keywords:** *Pseudomonas aeruginosa*, β-lactam, β-lactam resistance, β-lactamase, β-lactamase inhibitor, mutational mechanisms, evolution, ESBL, OXA, BEL, ceftazidime/avibactam

## Abstract

The emergence of ceftazidime/avibactam resistance in an extended-spectrum β-lactamase (ESBL)-producing *Pseudomonas aeruginosa* isolate during ceftazidime/avibactam therapy was investigated and the underlying mechanisms elucidated. Two sequential isolates recovered before and after the ceftazidime/avibactam treatment were characterized by antimicrobial susceptibility testing, whole-genome sequencing, and functional and biochemical analyses. The initial isolate was susceptible to ceftazidime/avibactam, whereas the later isolate developed resistance (increase in MIC from 2 to 32 mg/L), accompanied by collateral carbapenem susceptibility. Both isolates co-produced a novel BEL variant (designated BEL-5) and an OXA-2 enzyme, encoded by adjacent genes within a class one integron embedded in a 31,623-bp IncP plasmid. The resistant isolate harbored a previously undescribed OXA-2 variant, designated OXA-1381, carrying a W157L substitution in the Ω-loop. Cloning experiments demonstrated that OXA-1381 is an extended-spectrum variant conferring increased resistance to ceftazidime/avibactam and ceftolozane/tazobactam but decreased resistance to carbapenems. Kinetic analyses revealed a marked increase in the affinity and catalytic efficiency of OXA-1381 against ceftazidime and ceftolozane, while showing reduced hydrolytic activity against meropenem, consistent with the observed phenotypic trade-off. Inhibition assays showed similar susceptibility to avibactam for both enzymes, confirming that resistance was driven by enhanced hydrolysis rather than by impaired inhibition. The findings document the *in vivo* evolution of ceftazidime/avibactam resistance in an ESBL-producing *P. aeruginosa* background, mediated by structural modification of a narrow-spectrum OXA-2 enzyme. In addition, they highlight the potential of integron-borne genes encoding “silent” β-lactamases to evolve under antibiotic pressure and compromise the activity of last-line agents.

## INTRODUCTION

*Pseudomonas aeruginosa* is a leading cause of healthcare-associated infections and represents a major clinical challenge worldwide. Its impact is particularly severe in intensive care units and in immunocompromised patients, frequently causing life-threatening conditions, such as ventilator-associated pneumonia, bloodstream infections, and chronic respiratory diseases (1). As a high-priority ESKAPE pathogen (2–4), *P. aeruginosa* is characterized by high rates of resistance to key therapeutic agents, including antipseudomonal cephalosporins and carbapenems, which are associated with increased morbidity and mortality (5). The resistance is largely driven by its extensive, highly dynamic mutational resistome, which enables the selection of chromosomal mutations during therapy, leading to resistance mechanisms such as AmpC overproduction, upregulation of multidrug efflux systems, and loss or inactivation of the OprD porin (6). In addition to chromosomally encoded resistance mechanisms, *P. aeruginosa* has a remarkable capacity to acquire resistance determinants through horizontal gene transfer, including genes encoding extended-spectrum β-lactamases (ESBLs) and carbapenemases (7). Notably, these mechanisms often accumulate within single strains, particularly among globally disseminated high-risk clones, giving rise to difficult-to-treat resistance (DTR) phenotypes that further restrict therapeutic options (8).

In recent years, several novel and potent antipseudomonal agents have been introduced into clinical practice, including ceftazidime/avibactam, ceftolozane/tazobactam, imipenem/relebactam, and cefiderocol, helping to address the urgent need for effective treatments against DTR *P. aeruginosa* infections. Ceftolozane/tazobactam and imipenem/relebactam have expanded the therapeutic armamentarium against strains exhibiting β-lactam resistance driven by chromosomally encoded mechanisms, against which these agents remain highly active. Similarly, cefiderocol demonstrates remarkable stability against mutational resistance mechanisms but also represents one of the most effective options against metallo-β-lactamase (MBL)-producing *P. aeruginosa* (9). Ceftazidime/avibactam has shown comparable antimicrobial activity and clinical efficacy to ceftolozane/tazobactam against isolates with mutation-driven resistance, although its activity may be slightly more compromised in strains with upregulated MexAB-OprM-mediated efflux (10). Importantly, ceftazidime/avibactam is considered the treatment of choice for infections caused by class A ESBL-producing *P. aeruginosa*, including those harboring GES, VEB, PER, or BEL enzymes, which are often associated with resistance to ceftolozane/tazobactam and are prevalent in certain epidemiological settings (11). In this context, the recent ASPIRE-ICU study conducted in Europe reported that approximately 40% of β-lactam-resistant *P. aeruginosa* isolates from ICU patients carried an ESBL gene (12).

While several mechanisms have been associated with the development of ceftazidime/avibactam resistance in *P. aeruginosa*, this phenomenon has rarely been observed during the treatment of ESBL-producing *P. aeruginosa* infections. Notably, genes encoding ESBLs circulating in *P. aeruginosa* are frequently integron-borne and are therefore often associated with additional resistance determinants within gene cassettes (13, 14). Among these, genes encoding narrow-spectrum class D β-lactamases, such as OXA-2 and OXA-10, are commonly co-carried and represent some of the most prevalent β-lactamase genes identified in publicly available genomic databases (15). OXA-2- and OXA-10-like enzymes have traditionally been regarded as “silent passengers” within the *P. aeruginosa* phenotype, as their limited substrate profiles do not usually compromise the activity of broad-spectrum cephalosporins. However, the acquisition of amino acid substitutions in catalytically important regions can lead to extended-spectrum variants, which have been shown to confer cross-resistance to both ceftolozane/tazobactam and, more concerningly regarding treatment of ESBL-producing *P. aeruginosa*, ceftazidime/avibactam (16, 17).

This study reports the emergence of ceftazidime/avibactam resistance during treatment of a BEL-5 ESBL-producing *P. aeruginosa* isolate, driven by structural modification of a co-produced OXA-2 enzyme. It also provides functional and biochemical characterization of this novel OXA-2 variant, designated OXA-1381, which underlies the evolved resistance phenotype.

## RESULTS

### Antimicrobial susceptibility and phenotypic characterization of clinical isolates

Comparative antimicrobial susceptibility profiles of the two *P. aeruginosa* clinical isolates are summarized in Table 1. The initial isolate, *P. aeruginosa* ARGA00576, exhibited resistance to most clinically available antipseudomonal β-lactams, including piperacillin/tazobactam, ceftazidime, ceftolozane/tazobactam, aztreonam, cefepime, imipenem, meropenem, and meropenem/vaborbactam, but remained susceptible to ceftazidime/avibactam, aztreonam/avibactam, cefiderocol, imipenem/relebactam, and the investigational combinations cefepime/taniborbactam and cefepime/zidebactam. By contrast, the later isolate (*P. aeruginosa* ARGA00592) displayed an evolved resistance profile characterized by increases in the MICs of ceftazidime/avibactam (from 2 to 32 mg/L) and ceftolozane/tazobactam (from 8 to 64 mg/L). Notably, this isolate also exhibited increased susceptibility to carbapenems, with marked reductions in the MICs of imipenem/relebactam (from 2 to 0.25 mg/L), meropenem, and meropenem/vaborbactam (from 16 to 4 mg/L). No significant changes were observed for the remaining agents.

**TABLE 1:** Strain ID, sequence type, antimicrobial susceptibility profile, and mechanisms of β-lactam resistance in *P. aeruginosa* isolates ARGA00576 and ARGA00592

		MIC (mg/L)^*a*^*^,b^*														β-lactam resistance mechanisms^*c*^	
Strain ID	ST	P/T	CAZ	C/A	C/T	ATM	A/A	FEP	F/T	F/Z	FDC	IMP	I/R	MEM	M/V	Transferable	Mutational
		(R>16)	(R>8)	(R>8)	(R>4)	(R>16)	(R>16)	(R>8)	(R>8)	(R>8)	(R>2)	(R>4)	(R>2)	(R>8)	(R>8)		
ARGA00576	303	128	32	2	8	16	4	16	8	2	0.5	8	2	16	16	*bla*_BEL-5_ *bla*_OXA-2_	*oprD* (T71fs) *mexY* (V206L) *mexZ* (Q10*)
ARGA00592	303	128	64	32	64	16	4	16	8	2	1	4	0.25	4	4	*bla*_BEL-5_ *bla*_OXA-1381_	*oprD* (T71fs) *mexY* (V206L) *mexZ* (Q10*) *mpl* (M38fs)

^*a*^ P/T, piperacillin/tazobactam; CAZ, ceftazidime; C/A, ceftazidime/avibactam; C/T, ceftolozane/tazobactam; ATM, aztreonam; A/A, aztreonam/avibactam; FEP, cefepime; F/T, cefepime/taniborbactam; F/Z, cefepime/zidebactam; FDC, cefiderocol; IMP, imipenem; I/R, imipenem/relebactam; MEM, meropenem; M/V, meropenem/vaborbactam.

^*b*^ EUCAST v.16.0 breakpoints indicated.

^*c*^ fs, frameshift; *, premature stop codon. Mutations are listed according to their position in the *P. aeruginosa* PAO1 reference genome.

Phenotypic screening assays showed the following, for both isolates: (i) negative results in cloxacillin inhibition tests using ceftazidime, suggesting that cephalosporin resistance was not associated with AmpC hyperproduction; (ii) positive synergy with clavulanic acid, indicating the presence of an ESBL; and (iii) negative results in carbapenemase screening assays, supporting the absence of acquired carbapenemases and suggesting that carbapenem resistance was likely driven by chromosomal mechanisms. However, these assays did not provide any insights into the mechanisms underlying the increased ceftazidime/avibactam resistance observed in *P. aeruginosa* ARGA00592.

### Mechanisms of β-lactam resistance

**Fig 1:**
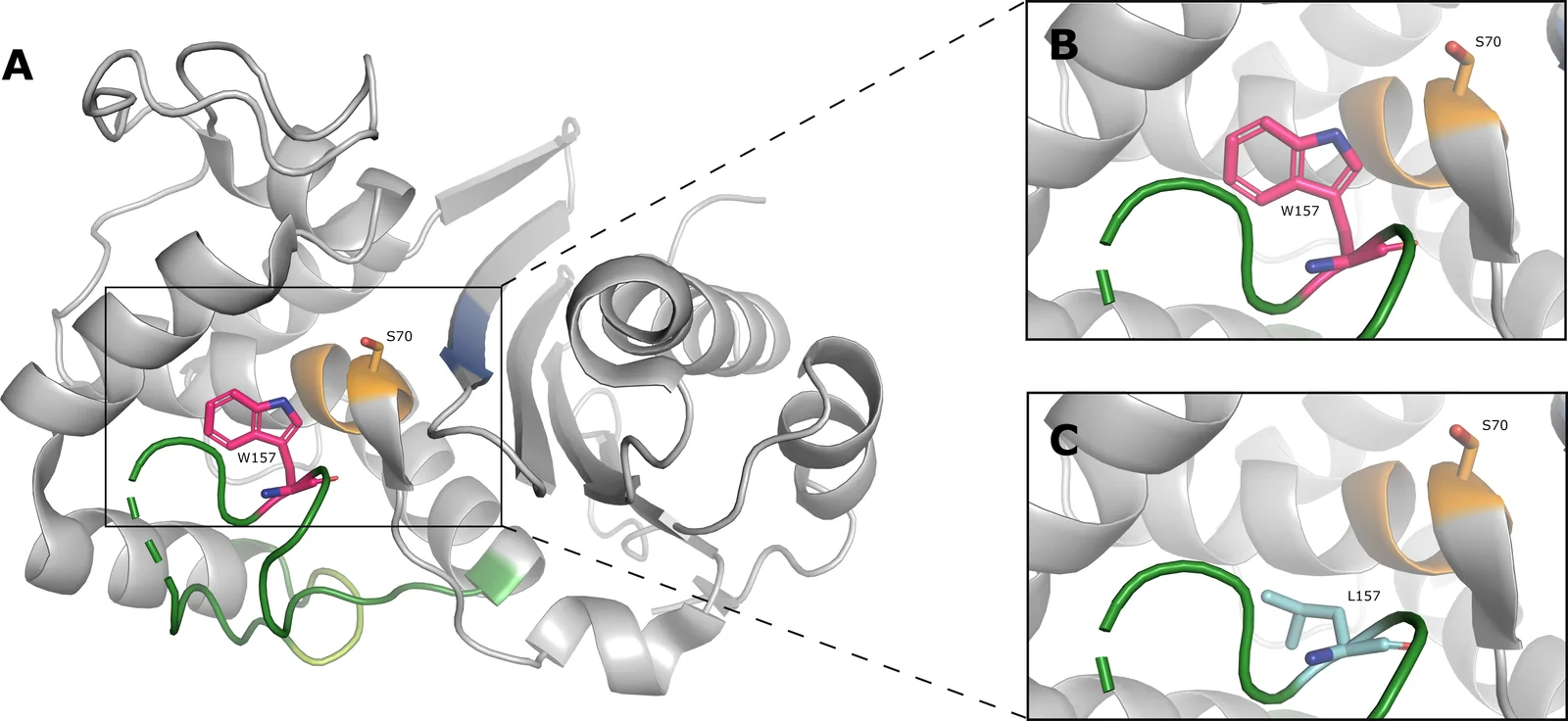
Structural model of OXA-2 highlighting the location of residue W157 within the Ω-loop. Structural visualization and *in silico* amino acid substitution were performed using PyMOL v3.1.8 based on the published OXA-2 crystal structure (PDB ID: 1K38, 1.50 Å). (**A**) Overall structure of OXA-2. Conserved catalytic motifs are highlighted as follows: the STFK motif is shown in orange, with the catalytic serine displayed in stick representation; the KTG motif is shown in dark blue; and the Ω-loop is shown in green, including the conserved YGN motif in light green. Residue W157, located within the Ω-loop, is shown in magenta in stick representation. (**B**) Enlarged view of the region surrounding W157 in the wild-type OXA-2 enzyme, illustrating the position of this highly conserved aromatic residue within the Ω-loop. (**C**) Enlarged view of the same region after *in silico* substitution of W157 by L157. The leucine residue is displayed in cyan in stick representation, illustrating the replacement of the bulky aromatic tryptophan by a smaller aliphatic residue within the Ω-loop.

The presence of acquired β-lactamase genes in both isolates was identified by whole-genome sequencing (WGS) (Table 1). Both isolates produced a previously undescribed A267E variant of the class A ESBL BEL-1, designated BEL-5, which accounts for the ESBL phenotype observed in double-disk synergy tests with clavulanic acid. In addition, both carried OXA-2-like β-lactamases. However, while *P. aeruginosa* ARGA00576 harbored a wild-type OXA-2 enzyme, the ceftazidime/avibactam-resistant *P. aeruginosa* isolate ARGA00592 carried a previously undescribed OXA-2 variant with a W157L substitution in the Ω-loop, designated OXA-1381. The location of residue W157 within the Ω-loop and the *in silico* W157L substitution are illustrated in Fig. 1.

Regarding chromosomally encoded resistance mechanisms, both isolates shared inactivating alterations in OprD and MexZ, as well as a conservative amino acid substitution in MexY. The *P. aeruginosa* ARGA00592 isolate also exhibited a truncation in Mpl, a ligase involved in peptidoglycan recycling (18). Loss-of-function mutations in *mpl* have been associated with *ampC* overexpression (19) and reduced susceptibility to β-lactams in *P. aeruginosa* clinical isolates (20).

### Genetic support of β-lactamases

**Fig 2:**
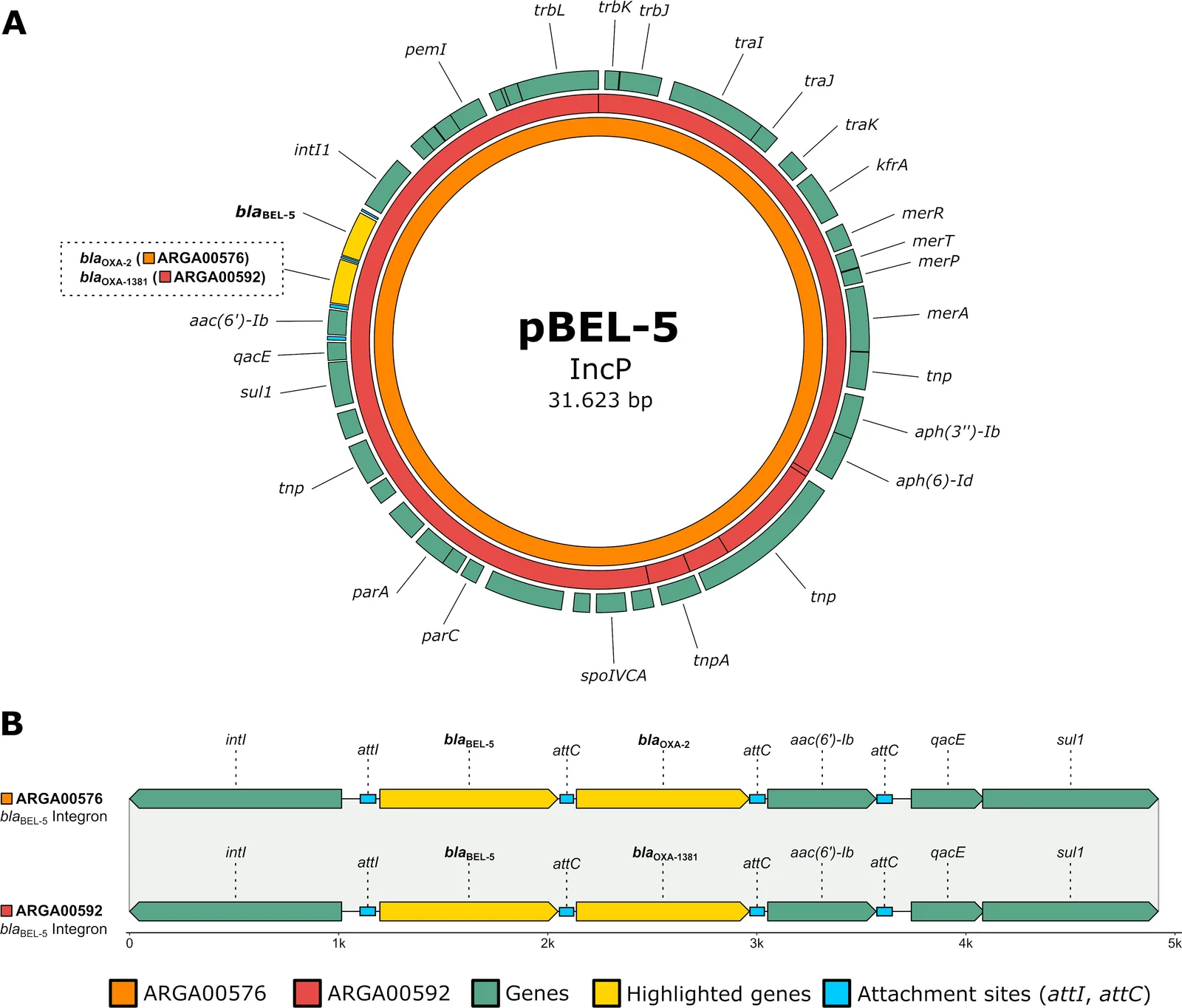
Comparative analysis of the *bla*_BEL-5_-carrying pBEL-5 plasmids identified in *P. aeruginosa* isolates ARGA00576 and ARGA00592. (**A**) Circular comparison of the IncP plasmids from isolates ARGA00576 and ARGA00592, showing their overall genetic content. (**B**) Detailed organization of the class one integron carrying *bla*_BEL-5_, including gene orientation, the integrase (*intI1*), *attI* and *attC* recombination sites, and associated resistance genes.

Long-read sequencing enabled the complete circularization of the genetic platform harboring the β-lactamase genes. Both the *bla*_BEL-5_ and *bla*_OXA-2-like_ genes were located on a 31,623-bp IncP-type plasmid present in both isolates (Fig. 2). These genes were located adjacently within a class 1 integron, with *bla*_OXA-2_ or *bla*_OXA-1381_ located immediately downstream of the *bla*_BEL-5_ gene cassette. In addition to β-lactam resistance determinants, the plasmid carried five additional resistance genes (*aph(3″)-Ib*, *aph (6)-Id*, *sul1*, *qacE,* and *aac(6′)-Ib*) and a mercury resistance operon. Furthermore, the plasmid encoded transfer-associated genes, and *in silico* analysis using MOB-suite predicted it to be mobilizable, although not conjugative.

### Contribution of BEL and OXA β-lactamases to β-lactam resistance

To assess the contribution of the β-lactamases identified in the clinical isolates to β-lactam resistance in *P. aeruginosa*, the *bla*_BEL-1_, *bla*_BEL-5_, *bla*_OXA-2_, and *bla*_OXA-1381_ genes were cloned and produced in the *P. aeruginosa* PAO1 background. Comparative MIC data for the resulting transformants are shown in Table 2. Production of BEL-1 and BEL-5 conferred an ESBL phenotype, characterized by increased MICs of broad-spectrum cephalosporins (including ceftolozane/tazobactam) and aztreonam, while susceptibility to carbapenems was maintained. Both enzymes displayed similar MIC profiles, although BEL-5 conferred slightly higher resistance to ceftazidime than BEL-1, with no significant differences observed for the remaining β-lactams or β-lactam/β-lactamase inhibitor combinations. Production of OXA-2 conferred the expected phenotype of a narrow-spectrum class D β-lactamase, without affecting susceptibility to broad-spectrum cephalosporins or carbapenems. By contrast, production of the W157L variant OXA-1381 resulted in a marked increase in MICs of ceftazidime/avibactam and ceftolozane/tazobactam (from 2 to 8 mg/L and from 0.5 to 8 mg/L, respectively), confirming the role of this variant in the ceftazidime/avibactam-resistant phenotype. Consistent with observations in the clinical isolates, meropenem MICs in the OXA-1381 transformant decreased to wild-type levels.

**TABLE 2:** Antimicrobial susceptibility profile for *P. aeruginosa* PAO1 complemented with recombinant plasmids encoding *bla*_BEL-1_, *bla*_BEL-5_, *bla,*_OXA-2_ and *bla*_OXA-1381_ genes

Strain	MIC (mg/L)^*a*^*^,b^*													
	P/T	CAZ	C/A	C/T	ATM	A/A	FEP	F/T	F/Z	FDC	IMP	I/R	MEM	M/V
	(R>16)	(R>8)	(R>8)	(R>4)	(R>16)	(R>16)	(R>8)	(R>8)	(R>8)	(R>2)	(R>4)	(R>2)	(R>8)	(R>8)
*P. aeruginosa* PAO1	4	2	2	0.5	4	4	2	2	1	0.125	1	0.25	0.5	0.5
PAO1 + BEL-1	512	16	2	8	32	4	16	2	2	1	1	0.25	0.5	0.5
PAO1 + BEL-5	256	64	4	16	64	4	32	2	2	1	1	0.25	0.5	0.5
PAO1 + OXA-2	4	4	2	0.5	4	4	2	2	1	1	1	0.25	2	2
PAO1 + OXA-1381	4	16	8	8	4	4	2	2	1	0.5	1	0.25	0.25	0.25

^*a*^ P/T, piperacillin/tazobactam; CAZ, ceftazidime; C/A, ceftazidime/avibactam; C/T, ceftolozane/tazobactam; ATM, aztreonam; A/A, aztreonam/avibactam; FEP, cefepime; F/T, cefepime/taniborbactam; F/Z, cefepime/zidebactam; FDC, cefiderocol; IMP, imipenem; I/R, imipenem/relebactam; MEM, meropenem; M/V, meropenem/vaborbactam.

^*b*^ EUCAST v.16.0 breakpoints indicated.

### Comparative biochemical properties of OXA-2 and OXA-1381 β-lactamases

To assess the biochemical impact of the W157L substitution on OXA-2, purified OXA-2 and OXA-1381 enzymes were subjected to steady-state kinetic analyses (Table 3). OXA-2 exhibited very low catalytic efficiency against cephalosporins, with minimal hydrolysis of both ceftazidime and ceftolozane. By contrast, OXA-1381 displayed markedly increased affinity for these substrates, as evidenced by substantial reductions in *K*_M_ values. Notably, the *K*_M_ for ceftazidime decreased by approximately three orders of magnitude (~3,000-fold), while a ~60-fold reduction was observed for ceftolozane. This enhanced substrate recognition translated into a pronounced increase in catalytic efficiency (*k*_cat_/*K*_M_ values) for both cephalosporins. For ceftazidime, the high *K*_M_ of OXA-2 precluded determination of *k*_cat_, whereas OXA-1381 exhibited measurable turnover (*k*_cat_ = 0.129 s^-1^), consistent with its acquired ceftazidime-hydrolyzing activity. Conversely, OXA-1381 showed reduced catalytic efficiency against meropenem, with a decrease in *k*_cat_/*K*_M_ from 6.51 to 1.57 mM^-1^ s^-1^, indicating a trade-off associated with diminished carbapenem hydrolysis. Inhibition assays revealed similar IC_50_ values for avibactam against both enzymes (Table 4), whereas OXA-1381 displayed moderately increased IC_50_ values for tazobactam, suggesting a limited impact on inhibitor susceptibility.

**TABLE 3:** Steady-state kinetic parameters of OXA-2 and OXA-1381 β-lactamases for representative substrates

	OXA-2			OXA-1381			Relative *k*_cat_/*K*_M_ ratio for OXA-1381/OXA-2
	*K*_M_ (μM) ±SD	*k*_cat_ (s^-1^) ±SD	*k*_cat_/*K*_M_ (mM^-1^ s^-1^)	*K*_M_ (μM) ±SD	*k*_cat_ (s^-1^) ±SD	*k*_cat_/*K*_M_ (mM^-1^ s^-1^)	
Nitrocefin	25.860 ± 5.118	54.166 ± 4.812	2,122.892 ± 216.089	31.833 ± 7.024	9.900 ± 0.367	319.802 ± 61.926	0.151
Ceftazidime	6,408.784 ± 690.283	n.d.^*a*^	n.d.^*a*^	1.988 ± 0.256	0.129 ± 0.013	64.657	n.d.^*a*^
Ceftolozane	640.237 ± 62.136	0.253 ± 0.031	0.396	9.838 ± 0.576	0.695 ± 0.102	70.598	176.496
Meropenem	0.018 ± 0.002	0.121 ± 0.034	6,514.740	0.009 ± 0.002	0.014 ± 0.003	1,570.215	0.241

^*a*^ n.d., not determined.

**TABLE 4:** Determination of the 50% inhibitory concentrations of β-lactamase inhibitors avibactam and tazobactam against OXA-2 and OXA-1381 β-lactamases

	IC_50_ (μM) ±SD	
	OXA-2	OXA-1381
Avibactam	0.587 ± 0.068	0.635 ± 0.095
Tazobactam	0.004 ± 0.001	0.049 ± 0.011

## DISCUSSION

Ceftazidime/avibactam is widely regarded as a cornerstone therapy for the treatment of DTR infections caused by non-MBL-producing *P. aeruginosa*, largely due to its potent activity against strains showing AmpC-mediated resistance to antipseudomonal cephalosporins (1). In addition, it represents a valuable therapeutic option for infections caused by strains producing class A β-lactamases, including ESBLs (e.g., BEL, VEB, PER, and GES-1) and some carbapenemases (e.g., KPC and GES-5), against which other β-lactam/β-lactamase inhibitor combinations, such as ceftolozane/tazobactam, imipenem/relebactam, and meropenem/vaborbactam, may show limited activity (7, 17, 21). Resistance to ceftazidime/avibactam in *P. aeruginosa* lacking acquired β-lactamases is most commonly driven by structural modifications in AmpC, particularly amino acid substitutions within the Ω-loop (22). In some other cases, resistance may also be driven by the combined effects of additional mechanisms, such as MexAB-OprM overproduction or alterations in PBP3 (e.g., R504C, F533L) (23). In strains producing broad-spectrum class A β-lactamases with carbapenemase activity, resistance has been associated with Ω-loop substitutions that enhance ceftazidime hydrolysis, as described for both GES- and KPC-type enzymes (24–26).

In this context, our findings expand current understanding by documenting the first case, to our knowledge, of ceftazidime/avibactam resistance emerging during targeted therapy of an ESBL-producing *P. aeruginosa* infection. Notably, and contrary to expectations, resistance did not arise through further optimization of the ESBL responsible for baseline cephalosporin resistance (in this case BEL-5) but was driven instead by the evolution of a co-produced OXA-2 enzyme not initially contributing to the resistance phenotype. This observation highlights the capacity of secondary, seemingly inconsequential, β-lactamases to act as evolutionary reservoirs for resistance under antibiotic pressure. Importantly, the integron- and plasmid-borne nature of these β-lactamase genes further amplifies the clinical relevance of this mechanism, as such genetic platforms facilitate co-selection and horizontal dissemination, raising concern about the potential spread of these resistance determinants among clinical *P. aeruginos*a strains (27). According to previous reports, *bla*_OXA-2-like_ genes are frequently found within gene cassettes associated with a wide range of clinically relevant broad-spectrum β-lactamase genes, including *bla*_GES_, *bla*_PER_, *bla*_VIM_, and *bla*_IMP_ (28, 29). In this study, we identified a *bla*_OXA-2_ gene co-located with a novel *bla*_BEL-5_ allele. Notably, BEL-5 expands the limited repertoire of BEL variants, which, in contrast to other class A ESBLs circulating in non-fermenters, such as PER (18 variants), GES (69 variants), and VEB (42 variants), have undergone relatively limited diversification (30). The E267A substitution identified in BEL-5 appears to have minimal impact on the substrate profile of BEL-1. To date, only the BEL-2 variant, carrying an F156L substitution, has been shown to exhibit enhanced cephalosporin-hydrolyzing activity relative to BEL-1 (31). None of the BEL variants described so far have been associated with resistance to ceftazidime/avibactam.

The role of narrow-spectrum oxacillinases, such as OXA-2 and OXA-10, which are widely distributed in *P. aeruginosa*, has traditionally been considered of limited clinical relevance. Even following the description of the first extended-spectrum OXA-2 variant, OXA-15 (D149G relative to OXA-2) (32), their impact remained secondary, largely due to the intrinsic ability of *P. aeruginosa* to develop resistance to classical cephalosporins through AmpC hyperproduction, and the predominance of more clinically significant ESBLs, such as VEB-1 and PER-1. However, the introduction of newer β-lactam/β-lactamase inhibitor combinations, including ceftolozane/tazobactam and ceftazidime/avibactam, has reshaped the clinical relevance of these enzymes. Under this therapeutic pressure, OXA-2- and OXA-10-like enzymes can act as evolutionary substrates, acquiring amino acid alterations that confer cross-resistance to both agents (33). This phenomenon has been documented for OXA-2-derived variants, such as OXA-539 (34) and OXA-681 (35), as well as for OXA-10 derivatives that emerge during therapy, including OXA-14, OXA-794, OXA-795, and OXA-824 (36). Although the growing number of reports suggest that the *in vivo* selection of mutations in narrow-spectrum OXA enzymes may represent an underestimated pathway for the evolution of resistance in *P. aeruginosa*, the global prevalence and clinical impact of such evolutionary events remain unknown.

According to the β-lactamase database (30) (accessed 10 April 2026), 32 OXA-2 variants have been described to date. To our knowledge, OXA-1381 is the first to carry a W157L substitution, although other substitutions at this position have been reported, such as OXA-210 (W157C) and OXA-543 (W157G) (37). The functional importance of this tryptophan residue is underscored by its high conservation across diverse class D β-lactamases, including OXA-1, OXA-10, OXA-23, and OXA-48, as described in the recently available SAND scheme (38). Structural modeling based on the published OXA-2 crystal structure (Fig. 1) showed that W157 is located within the Ω-loop, a key structural element of class D β-lactamases involved in substrate accommodation at the active site. The W157L substitution replaces a bulky aromatic residue with a smaller aliphatic amino acid, likely altering the local architecture of the Ω-loop and thereby affecting substrate recognition and positioning.

Here, we provide experimental evidence linking the W157L substitution to the ceftazidime/avibactam-resistant phenotype. Our biochemical analyses demonstrate that the W157L substitution leads to a substantial increase in catalytic efficiency toward cephalosporins, primarily driven by enhanced substrate affinity. Notably, similar effects have been described for the OXA-681 variant, which carries the ΔI158E159K alteration within the Ω-loop, adjacent to W157, and likewise exhibits increased catalytic efficiency associated with decreased *K*_M_ values (35). These findings highlight the Ω-loop as a critical evolutionary hotspot in OXA-2 enzymes, affecting cephalosporin hydrolysis without significantly compromising susceptibility to β-lactamase inhibitors. Interestingly, previous studies of ceftazidime/avibactam-resistant KPC variants have shown that such pronounced reductions in *K*_M_, particularly when accompanied by a reduction in *k*_cat_, may not exclusively reflect enhanced catalytic efficiency. In KPC-2 D179N, ceftazidime forms a long-lived acyl-enzyme intermediate that remains trapped in the active site, leading to a shift in the kinetic behavior of the enzyme (39). However, whether the same mechanism contributes to the altered kinetic behavior of OXA-1381 should be determined in future dedicated studies.

Notably, from a therapeutic perspective, cefiderocol retained activity against both parental and evolved isolates. In addition, carbapenems, particularly the imipenem/relebactam combination, which exhibited collateral susceptibility in the evolved strain, may represent a potential rescue therapy. However, when wild-type narrow-spectrum OXA enzymes are present in clinical *P. aeruginosa* isolates, strategies aimed at preventing the risk of resistance development should be applied. Such strategies include PK/PD-guided dosing and also maintaining high antibiotic concentrations by prolonged or continuous β-lactam infusion, as recently demonstrated in a hollow-fiber infection model (40).

In conclusion, we describe the emergence of ceftazidime/avibactam resistance in an ESBL-producing *P. aeruginosa* strain, mediated by the evolution of a co-produced narrow-spectrum OXA-2 β-lactamase into an extended-spectrum variant. These findings highlight the capacity of integron-borne “silent” β-lactamases to evolve under therapeutic pressure and compromise the activity of last-line agents, underscoring the need for continued surveillance and a deeper mechanistic understanding of resistance evolution in this high-risk pathogen.

## MATERIALS AND METHODS

### Clinical strains

The *P. aeruginosa* isolate ARGA00576 was recovered from a surveillance pharyngeal swab obtained from a patient 24 h after admission to the Intensive Care Unit (ICU) of the Complexo Hospitalario Universitario A Coruña (A Coruña, Spain) on 3 March 2025. After the patient developed respiratory deterioration, a bronchial secretion sample collected on March 12 yielded a *P. aeruginosa* isolate with an identical antimicrobial susceptibility profile, and treatment with ceftazidime/avibactam was initiated. Following completion of therapy, another *P. aeruginosa* isolate (ARGA00592) was recovered from a surveillance pharyngeal swab collected April 1, after the patient had been transferred to the hospital ward.

### Phenotypic screening of resistance mechanisms

Phenotypic screening for β-lactam resistance mechanisms included cloxacillin inhibition tests with ceftazidime as the reporter substrate to assess AmpC hyperproduction (41), as well as double-disk synergy tests with antipseudomonal cephalosporins and clavulanic acid to evaluate ESBL production (42). The presence of carbapenemases was assessed using the β-Carba Test (Bio-Rad, CA, USA), the carbapenem inactivation method (CIM) (43), and the OKNVI lateral flow immunoassay (Coris BioConcept, Belgium).

### WGS and resistance genomics

The resistome of the *P. aeruginosa* isolates was characterized using WGS. Genomic DNA was extracted using the Wizard Genomic DNA Purification Kit (Promega, WI, USA). Both isolates (ARGA00576 and ARGA00592) were sequenced using an Illumina platform (Illumina Inc., CA, USA). To obtain a high-quality hybrid assembly for comparative analysis, the genome of the initial isolate (ARGA00576) was additionally sequenced using a PacBio long-read platform at an external facility (Macrogen Inc., Seoul, South Korea). Quality control of Illumina reads was performed using fastp (v0.23.2) (44), while long reads were filtered using filtlong (v0.2.1) (45). Host contamination was removed using BMTagger (v3.101) (46).

The *P. aeruginosa* isolate ARGA00576 was assembled using Flye (2.9-b1768) (47) following a long-read-first approach, whereas *P. aeruginosa* isolate ARGA00592 was assembled from Illumina short reads using Unicycler (v0.5.0) (48). Both assemblies were subsequently polished with Polypolish (49). Assembly quality was assessed for completeness and contamination using CheckM (v1.1.3) (50).

Putative open reading frames were annotated using Bakta (v1.7.0) (51). The sequence type (ST) was determined using the Center for Genomic Epidemiology (CGE) multilocus sequence typing (MLST) tool (52). Acquired resistance genes were identified using RGI (v5.2.0) in combination with the CARD database (v3.2.8) (53). The chromosomal mutational resistome was analyzed by variant calling with Snippy (v4.6.0) (54), using the *P. aeruginosa* PAO1 genome (NC_002516.2) as reference. Additionally, pairwise variant calling between the clinical isolates was performed with Snippy to identify additional chromosomal or plasmid variants that may have contributed to the observed phenotypic differences. Plasmid detection and prediction of mobilization and conjugation potential were performed using MOB-suite (v3.1.0) (55). Plasmid visualizations were generated using Brick (v0.4.1) (56). Class 1 integrons and their recombination sites (*attI* and *attC*) were identified using IntegronFinder (v2.0) (57).

### Structural visualization and *in silico* mutagenesis

Structural visualization was performed using the published crystal structure of OXA-2 (PDB ID: 1K38, 1.50 Å) in PyMOL (v3.1.8) (58). Residues were numbered according to the standard ambler numbering (SAND) scheme for class D β-lactamases (30). Catalytically relevant structural elements, including the STFK motif, the KTG motif, the Ω-loop, the YGN motif, and residue W157, were highlighted for visualization. The W157L substitution was introduced using the Mutagenesis Wizard tool implemented in PyMOL.

### Molecular cloning

The contribution of BEL-1, BEL-5, OXA-2, and OXA-1381 to β-lactam resistance was assessed by molecular cloning with the multicopy plasmid pUCP24, and *P. aeruginosa* PAO1 as the host strain. The *bla*_BEL-1_, *bla*_BEL-5_, *bla*_OXA-2_, and *bla*_OXA-1381_ genes were amplified from the clinical isolates using the following primers: (i) for BEL-like β-lactamases, BEL Fw-*Eco*RI (5′-GGCGGTTTTCATGGCTTGTTATG-3′) and BEL Rv-*Bam*HI (5′-GCCTAACGCTTGAGCCAAG-3′); and (ii) for OXA-2-like β-lactamases, OXA Fw-*Sac*I (5′-CGAGCTCGGTTGGGCATTAAGGAAAAGTTAA-3′) and OXA Rv-*Hind*III (5′-TCCAAGGCTTTTATCGCGCAGCGTCCGAG-3′).

Purified PCR products were digested and ligated into the pUCP24 plasmid following previously described procedures (59). Recombinant plasmids were first electroporated into *E. coli* TG1, and transformants were selected on LB agar plates supplemented with gentamicin (10 mg/L). After confirmation by Sanger sequencing, the constructs were electroporated into *P. aeruginosa* PAO1, and transformants were selected on LB agar plates containing gentamicin (30 mg/L).

### Antimicrobial susceptibility testing

Minimum inhibitory concentrations (MICs) were determined for both clinical isolates and *P. aeruginosa* PAO1 transformants. MICs were assessed in triplicate using the reference broth microdilution method in cation-adjusted Mueller-Hinton broth (CAMHB), in accordance with CLSI M100 guidelines (60). Quality control strains included *P. aeruginosa* ATCC 27853, *Klebsiella pneumoniae* ATCC 700603, *Escherichia coli* NCTC 13353, and *K. pneumoniae* ATCC BAA-2814.

The antimicrobial agents tested were piperacillin/tazobactam, ceftazidime, ceftazidime/avibactam, ceftolozane/tazobactam, aztreonam, aztreonam/avibactam, cefepime, cefepime/taniborbactam, cefepime/zidebactam, cefiderocol, imipenem, imipenem/relebactam, meropenem, and meropenem/vaborbactam. Tazobactam, avibactam, taniborbactam, and relebactam were tested at a fixed concentration of 4 mg/L, vaborbactam at 8 mg/L, and zidebactam at a 1:1 ratio with cefepime.

Cefiderocol susceptibility testing was performed in iron-depleted CAMHB, prepared according to CLSI M100 recommendations using BD BBL MH broth (60, 61). MIC results were interpreted according to the European Committee on Antimicrobial Susceptibility Testing (EUCAST) clinical breakpoints (v 16.0, https://www.eucast.org/clinical_breakpoints/). For antimicrobial combinations not yet approved, interpretation was based on the breakpoints of the β-lactam partner agent alone.

### Protein purification

The *bla*_OXA-2_ and *bla*_OXA-1381_ genes were cloned into the pGEX-6P-1 expression plasmid (Cytiva, MA, USA) using primers designed to exclude the signal peptide sequence, thereby preventing periplasmic targeting of the fusion protein and facilitating purification from the bacterial cytoplasm, as previously described (62). Amplification was performed with primers containing *Bam*HI and *Sma*I restriction sites: Fw-*Bam*HI (5′-CGCGGATCCGCGCAAGAAGGCACG-3′) and Rv-*Sma*I (5′-TCCCCCGGGGGATTATCGCGCAGCGTCC-3′).

PCR products were digested and ligated into the pGEX-6P-1 vector. The resulting constructs were electroporated into *E. coli* BL21, and transformants were selected on LB agar plates containing ampicillin (100 mg/L). Protein expression was induced with 0.1 mM isopropyl β-D-1-thiogalactopyranoside (IPTG) for 20 h at 25°C, yielding glutathione S-transferase (GST) fusion proteins with the OXA enzymes.

Fusion proteins were purified using the GST Gene Fusion System (Cytiva) according to the manufacturer’s instructions. The GST tag was removed by cleavage with PreScission protease (Cytiva), and the purity of the resulting proteins was confirmed by sodium dodecyl sulfate-polyacrylamide gel electrophoresis (SDS-PAGE), showing no detectable contaminating bands, consistent with an estimated purity of ≥99%.

### Steady-state kinetic parameters

Steady-state kinetic parameters of purified OXA-2 and OXA-1381 β-lactamases were determined spectrophotometrically at room temperature, as previously described (35, 62). Enzymatic reactions were carried out in 50 mM sodium phosphate buffer supplemented with 20 mM sodium bicarbonate (pH 7.2). Affinity constants (*K*_M_) for ceftazidime, ceftolozane, and meropenem were determined as *K*_i app_ in competitive inhibition assays using nitrocefin as a reporter substrate in a microplate spectrophotometer (Epoch 2, BioTek, VT, USA). First, *K*_M_ and *k*_cat_ parameters for nitrocefin were determined under initial-rate conditions and calculated using the Lineweaver-Burk linearization. These *K*_M_ values were used to select a saturating nitrocefin concentration (150 µM) for subsequent competitive inhibition assays. For competitive inhibition assays, purified enzymes were incubated with nitrocefin and increasing concentrations of each β-lactam. Initial rates for nitrocefin hydrolysis were calculated and used to obtain linear plots (1/V versus β-lactam concentration). *K*_i app (observed)_ values were obtained from linear regression analysis as the ratio of the y-intercept to the slope (Y_0_/slope). *K*_i app (observed)_ was then corrected using equation 1.


(1) $$K_{iapp(corrected)}=\frac{K_{iapp(observed)}}{\left[1+([NCF]/K_{M}(NCF)\right]}$$

When possible, catalytic rate constants (*k*_cat_) were determined in the Spectord 200 Plus spectrophotometer (Analytik Jena, Thuringia, Germany) by monitoring direct hydrolysis of each antibiotic under saturating substrate concentrations (well above *K*_M_) to ensure maximum reaction velocity (V_max_).

The following wavelengths and molar extinction coefficients (ε) were used: 482 nm (ε = 15,900 M^-1^ cm^-1^) for nitrocefin, 260 nm (ε = 8,660 M^-1^ cm^-1^) for ceftazidime, 254 nm (ε = 6,810 M^-1^ cm^-1^) for ceftolozane, and 297 nm (ε = 10,940 M^-1^ cm^-1^) for meropenem. Each kinetic parameter was determined from three independent experiments, with a standard deviation ≤30% of the mean. All kinetic plots together with the corresponding calculations of the steady-state kinetic parameters are provided in the Supplementary Material (Tables S1 through S10).

### Calculation of IC_50_ values

The inhibitory activity of avibactam and tazobactam against OXA-2 and OXA-1381 was evaluated using nitrocefin as a reporter substrate, as previously described (63). Purified enzymes were preincubated with increasing concentrations of each inhibitor for 10 min at room temperature, after which residual β-lactamase activity was determined by monitoring nitrocefin hydrolysis at 482 nm using a microplate spectrophotometer (Epoch 2). Initial velocities were determined from the linear phase of nitrocefin hydrolysis. The inverse of the initial velocity (1/V) was plotted against inhibitor concentration, and the data were fitted by linear regression. The IC_50_ value was calculated as shown in equation 2, where V_0_ is the initial velocity in the absence of inhibitor, and m and b correspond to the slope and *y*-intercept of the fitted regression line, respectively.


(2) $${IC}_{50}=\frac{2\times \left(1/V_{0}\right)-b}{m}$$

All measurements were performed in triplicate. IC_50_ linearization plots are provided in the Supplementary Material (Tables S11 through S14).

## Data Availability

The nucleotide sequences of the *bla*_BEL-5_ and *bla*_OXA-1381_ genes described in this study have been deposited in GenBank under accession numbers PV474612 and PV707973, respectively. The genome assemblies and raw sequencing reads from *P. aeruginosa* isolates ARGA00576 and ARGA00592 are available in the NCBI Sequence Read Archive (SRA) under BioProject accession number PRJNA1133624 (BioSamples SAMN51960744 and SAMN51960757).

## SUPPLEMENTAL MATERIAL

Supplemental Material OXA-1381 aac.00584-26-s0001.pdf Tables S1 to S14.

ASM does not own the copyrights to Supplemental Material that may be linked to, or accessed through, an article. The authors have granted ASM a non-exclusive, world-wide license to publish the Supplemental Material files. Please contact the corresponding author directly for reuse.
