# Supplementary material for: In vivo emergence of ceftazidime/avibactam resistance in Pseudomonas aeruginosa driven by OXA-1381, a novel W157L Ω-loop variant of OXA-2
Source: Antimicrob Agents Chemother. 2026 Aug 19;70(10):e00584-26. doi: 10.1128/aac.00584-26 (PMC13689465; doi:10.1128/aac.00584-26)
Supplement: Supplementary file 1 [file aac.00584-26-s0001.pdf]

Supplementary Material for

In vivo emergence of ceftazidime/avibactam resistance in *Pseudomonas aeruginosa* driven by OXA-1381, a novel W157L Ω-loop variant of OXA-2

Gloria Pérez-Rodríguez[1*], Lucía González-Pinto[1,2*], Pablo Aja-Macaya[1], Carlos Molina-Cáceres[1], Lucía Sánchez-Peña[1], Tania Blanco-Martín[1,2], Antonio Oliver[2,3], Germán Bou[1,2,4], Jorge Arca-Suárez[1,2#]

1- Servicio de Microbiología Clínica & Grupo de Investigación en Microbiología. Instituto de Investigación Biomédica de A Coruña (INIBIC), Complexo Hospitalario Universitario de A Coruña (CHUAC), SERGAS. Universidade da Coruña (UDC), A Coruña, Spain.

2- CIBER de Enfermedades Infecciosas (CIBERINFEC), Instituto de Salud Carlos III, Madrid, España.

3- Servicio de Microbiología, Servicio de Microbiología and Unidad de Investigación, Hospital Universitario Son Espases, Instituto de Investigación Sanitaria Illes Balears (IdISBa), Palma de Mallorca, Spain.

4- Departamento de Fisioterapia, Medicina y Ciencias Biomédicas, Universidade da Coruña, A Coruña, Spain.

* Gloria Pérez-Rodríguez and Lucía González-Pinto contributed equally to this work. Author order was determined by consensus among all authors, taking into account their respective contributions to the study.

# Corresponding author:

Dr. Jorge Arca-Suárez

e-mail: jorge.arca.suarez@sergas.es

Servicio de Microbiología Clínica & Grupo de Investigación en Microbiología. Instituto de Investigación Biomédica de A Coruña (INIBIC), Complexo Hospitalario Universitario de A Coruña (CHUAC), SERGAS. Universidade da Coruña (UDC), As Xubias S/N, 3º Planta, 15006, A Coruña, Spain

CIBER de Enfermedades Infecciosas (CIBERINFEC), Instituto de Salud Carlos III, Madrid, España

Phone: 0034679082502

Supplementary Material - Table of Contents

1. Table S1 .... S3
2. Table S2 .... S4
3. Table S3 .... S5
4. Table S4 .... S6
5. Table S5 .... S7
6. Table S6 .... S8
7. Table S7 .... S9
8. Table S8 .... S10
9. Table S9 .... S11
10. Table S10 .... S12
11. Table S11 .... S13
12. Table S12 .... S13
13. Table S13 .... S14
14. Table S14 .... S14

Table S1. Michaelis-Menten curves and corresponding Lineweaver-Burk plots for nitrocefin (NCF) hydrolysis by OXA-2. Results from three independent experiments are shown. For each experiment, the Michaelis-Menten curve and the corresponding Lineweaver-Burk linearization are presented together with the regression equation, coefficient of determination (R^2), and the calculated kinetic parameters (K_M, k_{cat} and k_{cat}/K_M).

Michaelis-Menten plots | **Lineweaver-Burk**

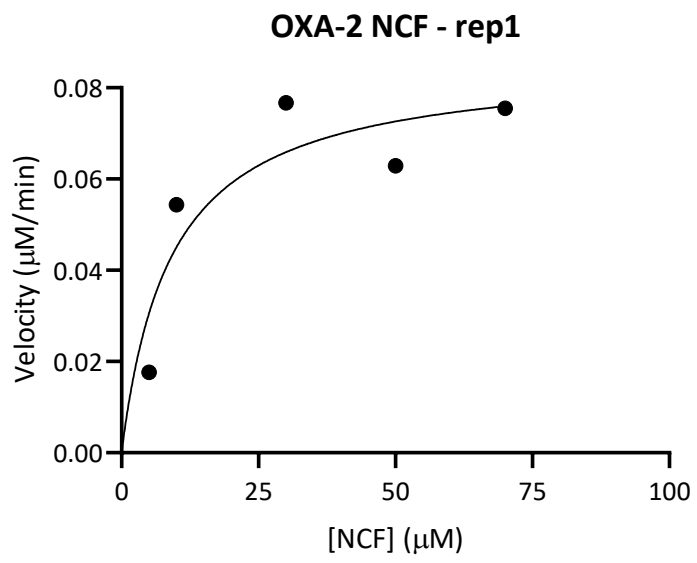

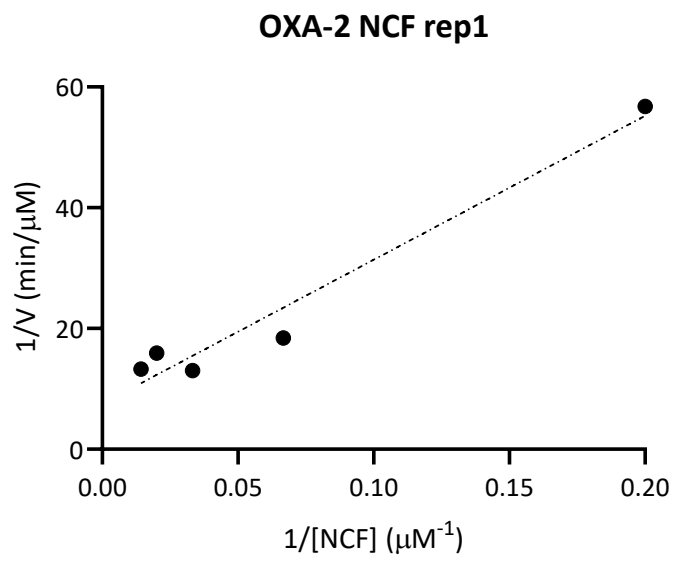

y = 238.494x + 7.529
R^2 = 0.963

K_M = 31.676 µM
k_{cat} = 59.359 s^{-1}
k_{cat}/K_M = 1873.967 mM^{-1} s^{-1}

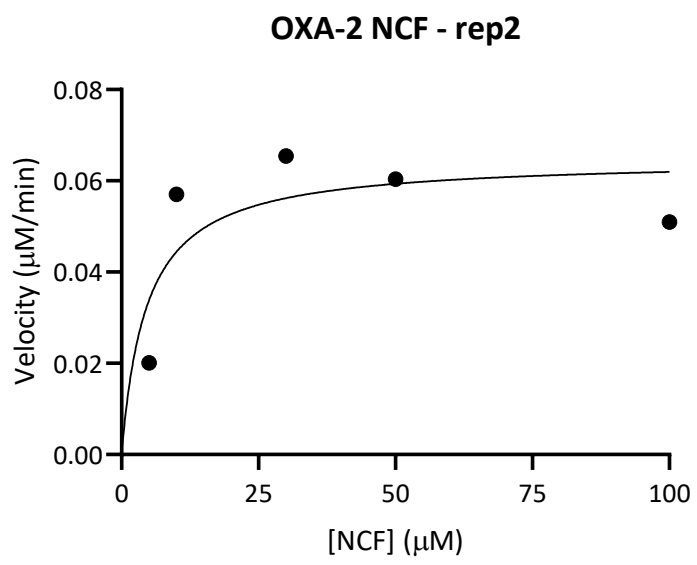

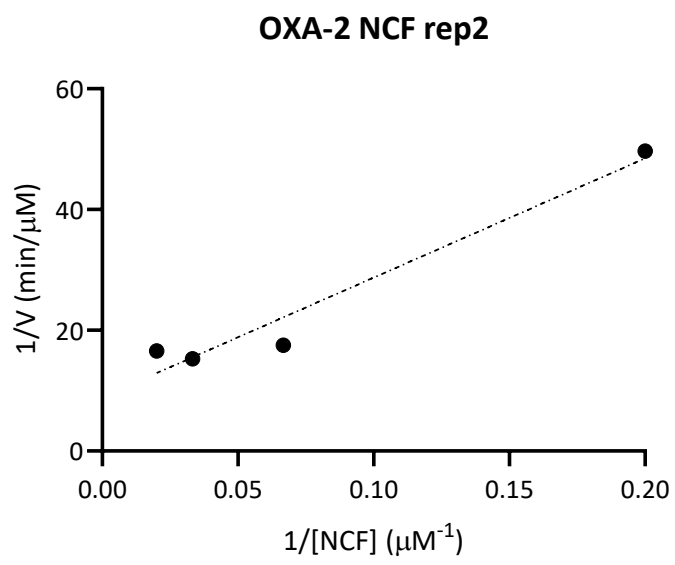

y = 197.561x + 8.964
R^2 = 0.957

K_M = 22.040 µM
k_{cat} = 49.859 s^{-1}
k_{cat}/K_M = 2262.235 mM^{-1} s^{-1}

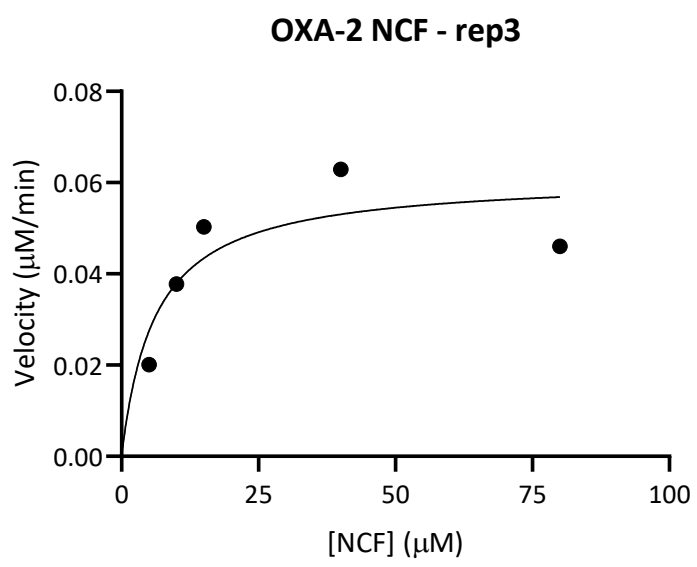

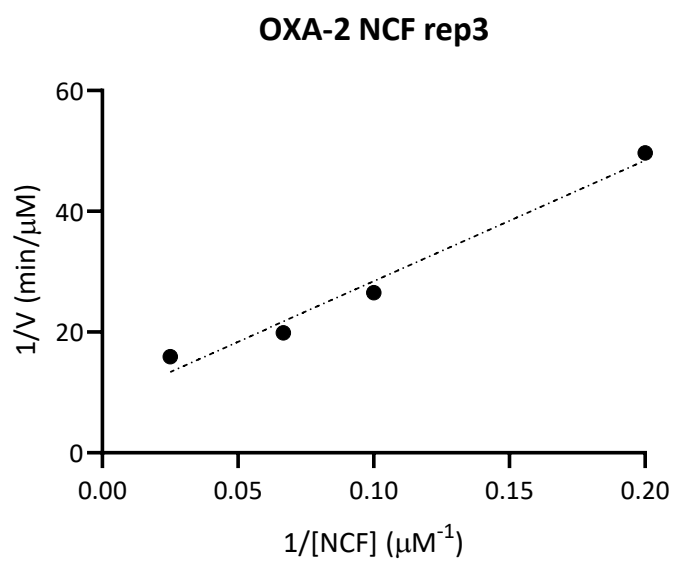

y = 200.195x + 8.388
R^2 = 0.978

K_M = 23.866 µM
k_{cat} = 53.281 s^{-1}
k_{cat}/K_M = 2232.474 mM^{-1} s^{-1}

Table S2. Competitive inhibition assays for the determination of the apparent inhibition constant ($K_{i\ app}$) of ceftazidime (CAZ) against OXA-2 using nitrocefin as the reporter substrate. Three independent experiments are shown. For each experiment, the linearization plot is presented together with the regression equation, coefficient of determination (R^2), and the calculated $K_{i\ app}$ values. Nitrocefin concentration was fixed at 150 µM.

Nitrocefin vs Ceftazidime	Linearization	
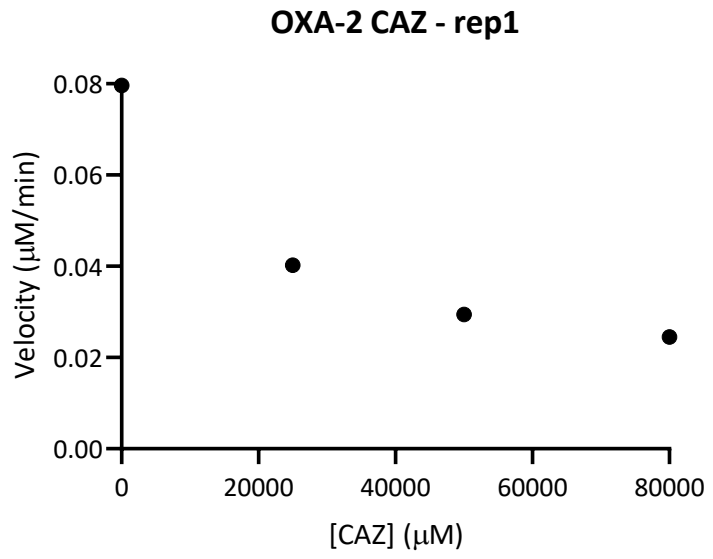	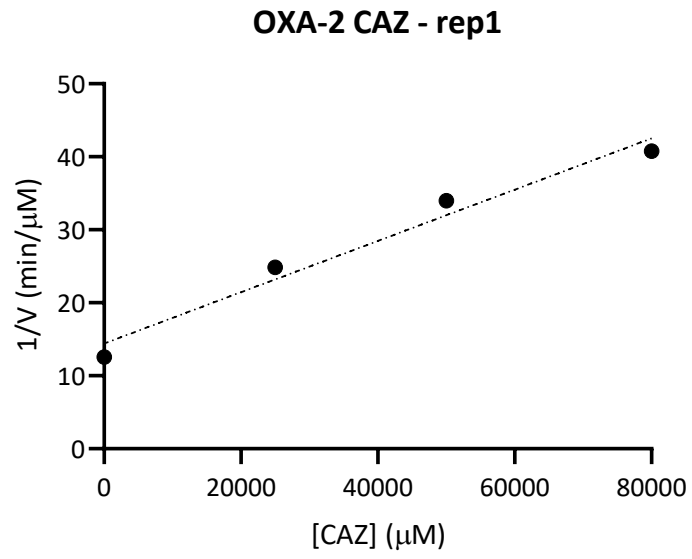	*y = 0.0004x + 14.424*<br>*R² = 0.971*<br>$K_{i\ app\ (observed)}$ = 41067.165 µM<br>$K_{i\ app\ (corrected)}$ = 6038.877 µM
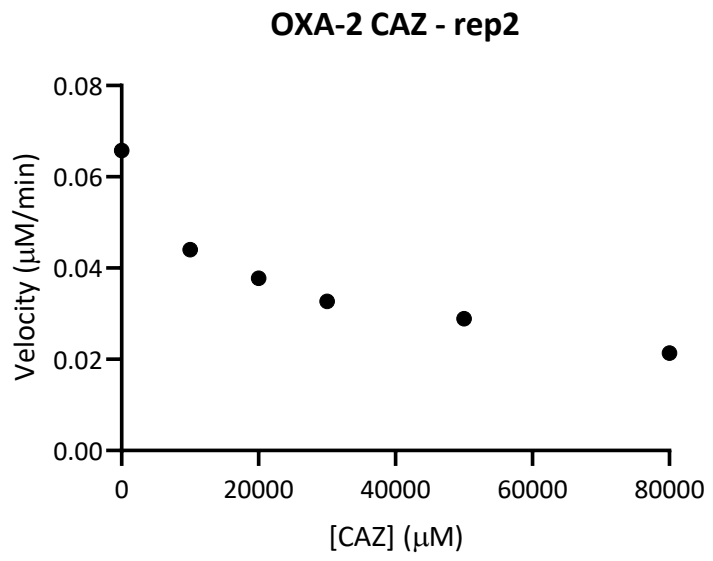	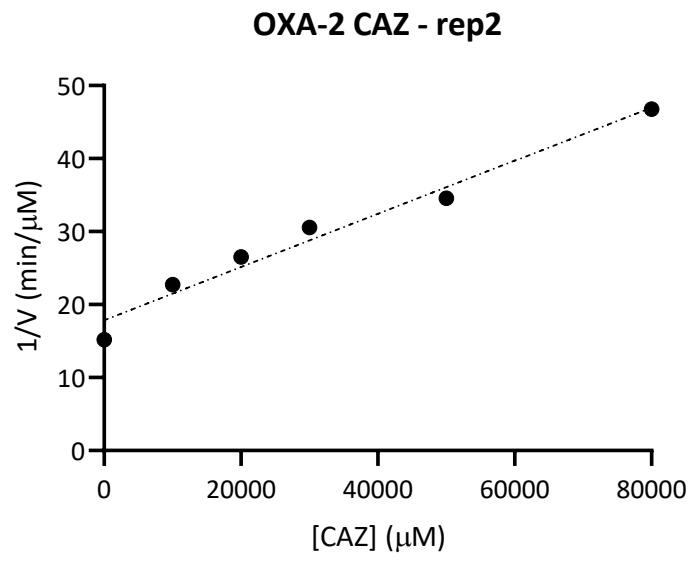	*y = 0.0004x + 17.850*<br>*R² = 0.973*<br>$K_{i\ app\ (observed)}$ = 48998.593 µM<br>$K_{i\ app\ (corrected)}$ = 7205.184 µM
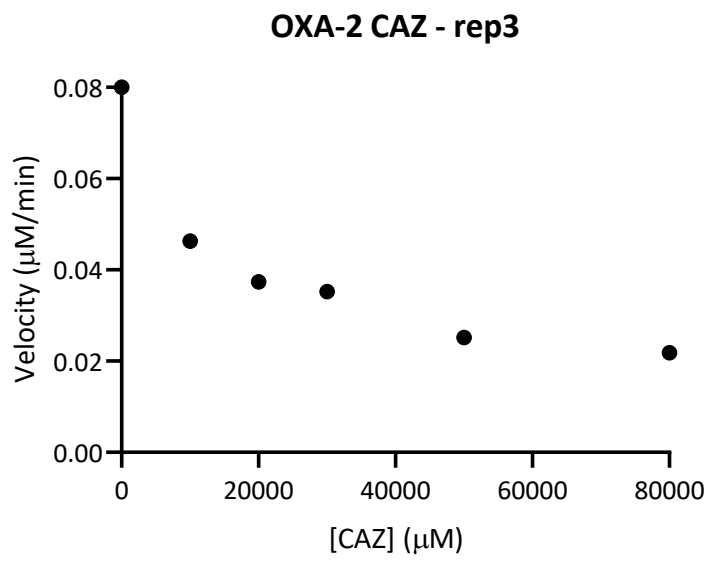	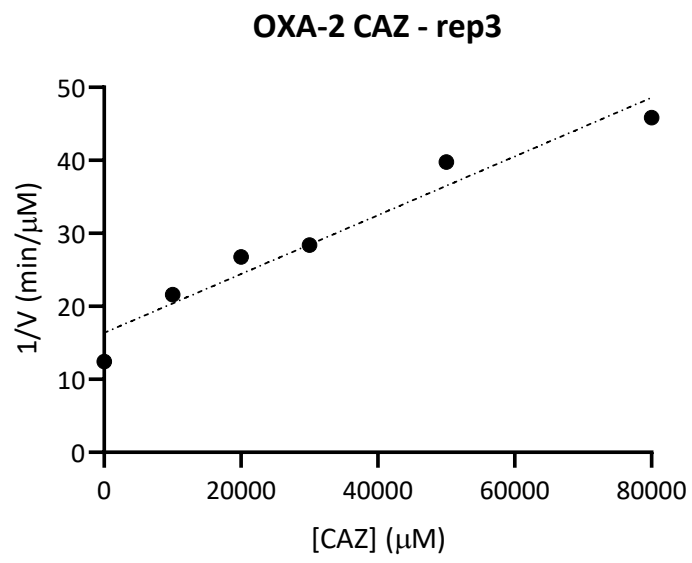	*y = 0.0004x + 16.381*<br>*R² = 0.945*<br>$K_{i\ app\ (observed)}$ = 40682.352 µM<br>$K_{i\ app\ (corrected)}$ = 5982.291 µM

Table S3. Competitive inhibition assays for the determination of the apparent inhibition constant ($K_{i\ app}$) of ceftolozane (COZ) against OXA-2 using nitrocefin as the reporter substrate. Three independent experiments are shown. For each experiment, the linearization plot is presented together with the regression equation, coefficient of determination (R^2), and the calculated $K_{i\ app}$ values. Nitrocefin concentration was fixed at 150 µM.

**Nitrocefin vs Ceftolozane**	**Linearization**	
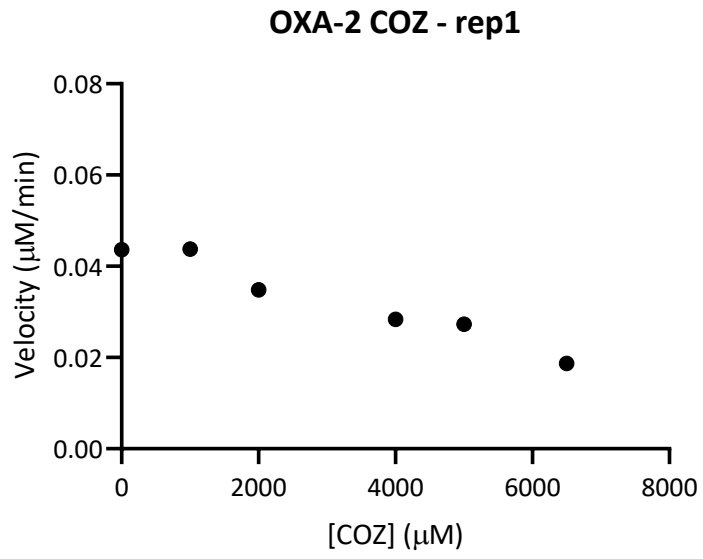	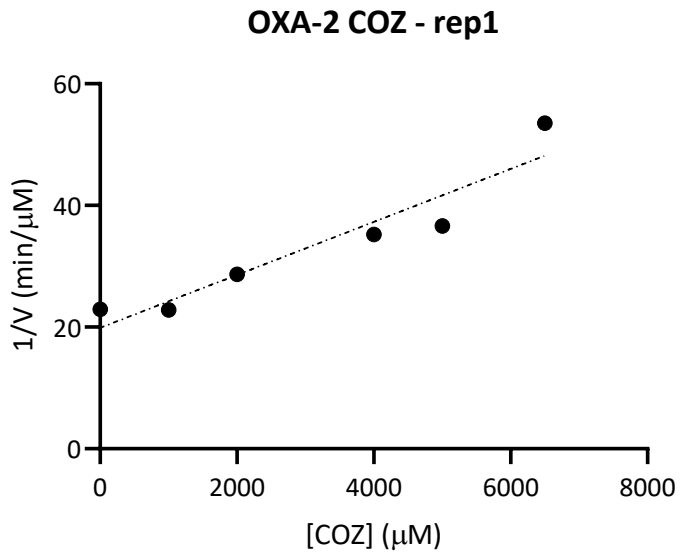	*y = 0.00435x + 19.874*<br>*R^2 = 0.895*<br>$K_{i\ app\ (observed)}$ = 4563.689 µM<br>$K_{i\ app\ (corrected)}$ = 671.085 µM
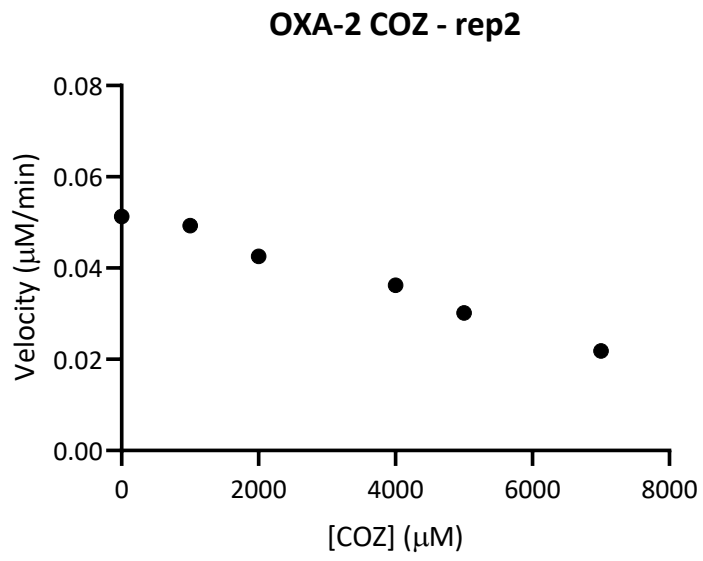	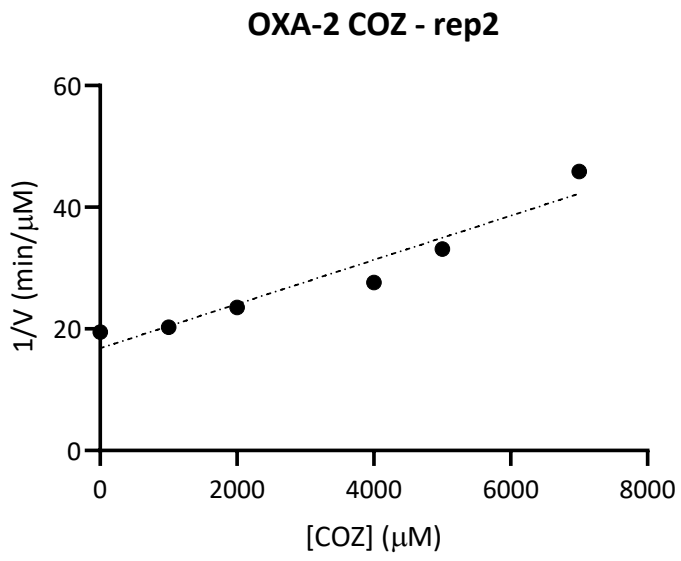	*y = 0.00363x + 16.813*<br>*R^2 = 0.924*<br>$K_{i\ app\ (observed)}$ = 4630.519 µM<br>$K_{i\ app\ (corrected)}$ = 680.912 µM
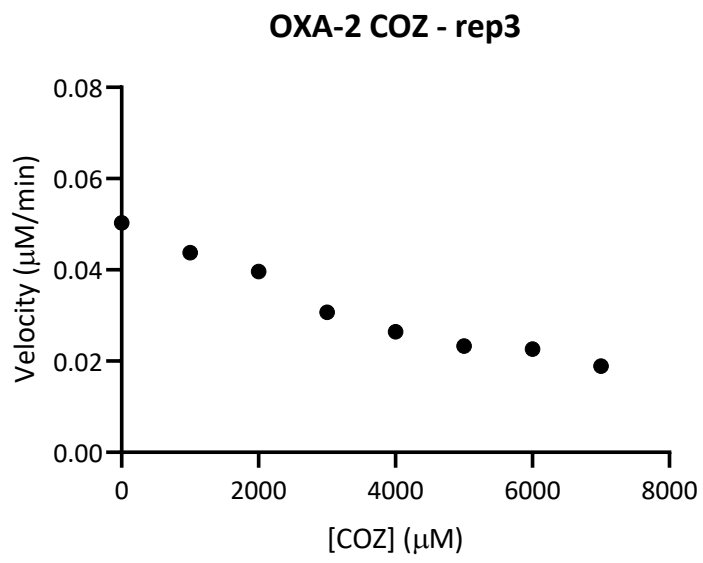	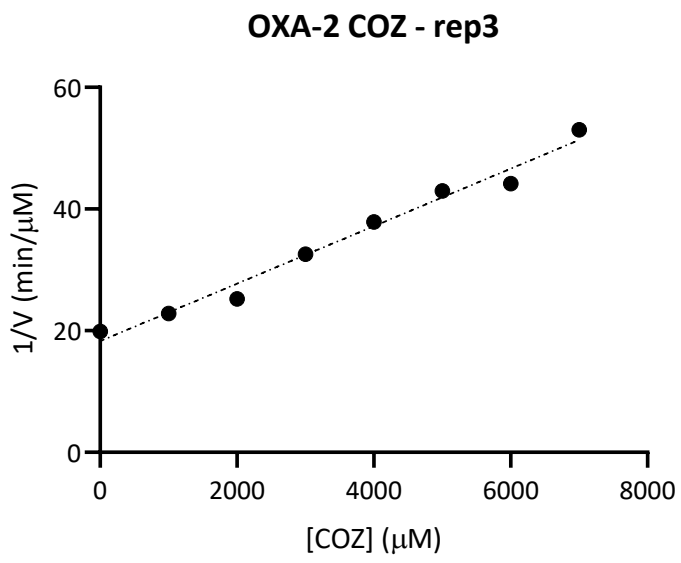	*y = 0.005x + 18.277*<br>*R^2 = 0.980*<br>$K_{i\ app\ (observed)}$ = 3867.507 µM<br>$K_{i\ app\ (corrected)}$ = 568.712 µM

Table S4. Competitive inhibition assays for the determination of the apparent inhibition constant ($K_{i\ app}$) of meropenem (MEM) against OXA-2 using nitrocefin as the reporter substrate. Three independent experiments are shown. For each experiment, the linearization plot is presented together with the regression equation, coefficient of determination (R^2), and the calculated $K_{i\ app}$ values. Nitrocefin concentration was fixed at 150 μM.

Nitrocefin vs Meropenem	Linearization	
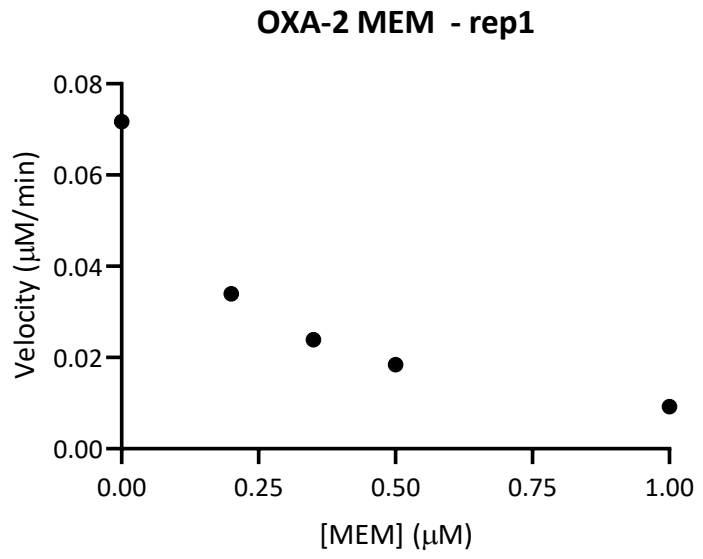	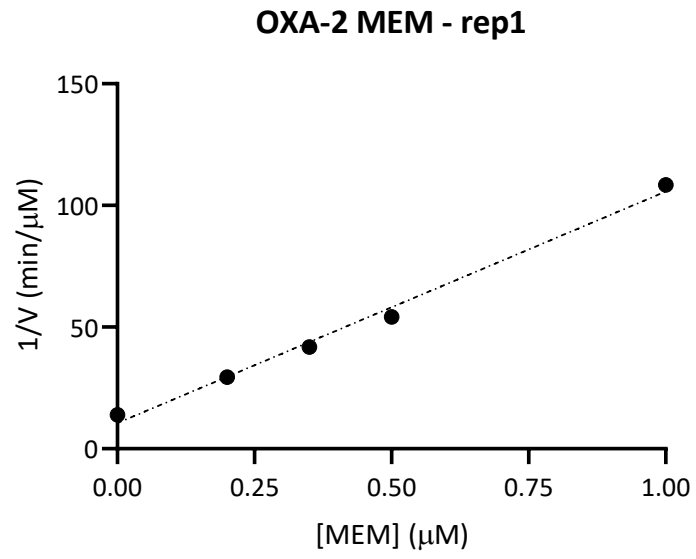	*y = 94.108x + 10.910*<br>*R² = 0.998*<br>$K_{i\ app\ (observed)}$ = 0.116 μM<br>$K_{i\ app\ (corrected)}$ = 0.017 μM
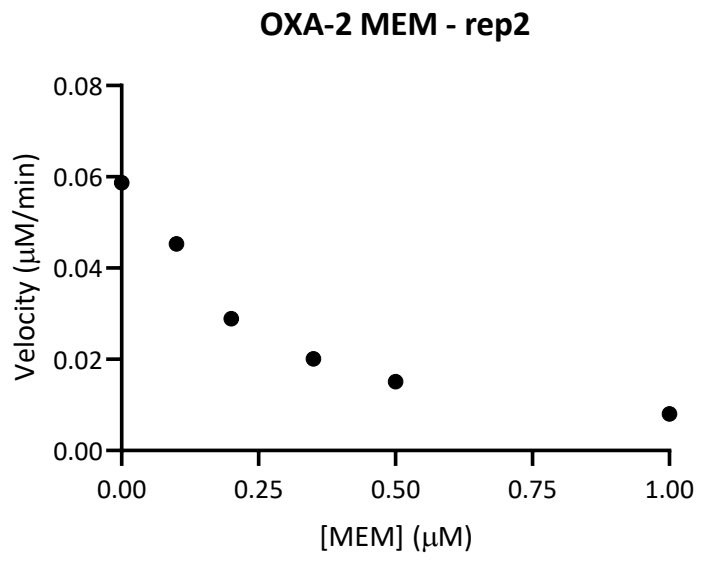	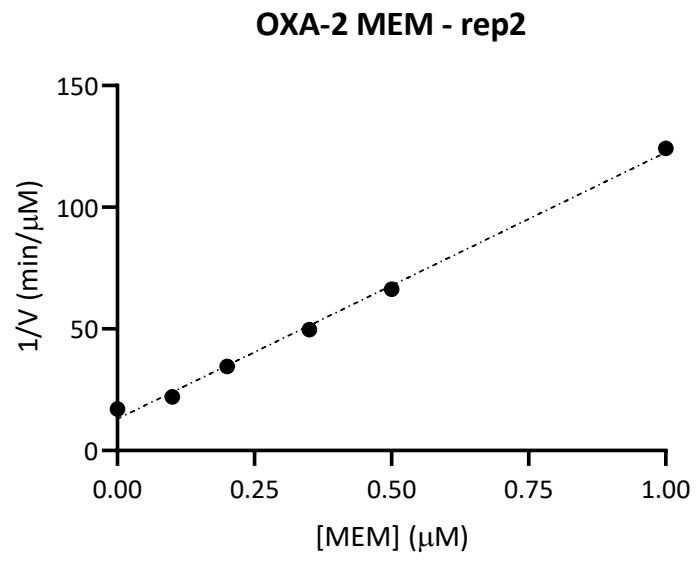	*y = 109.489x + 13.073*<br>*R² = 0.997*<br>$K_{i\ app\ (observed)}$ = 0.119 μM<br>$K_{i\ app\ (corrected)}$ = 0.018 μM
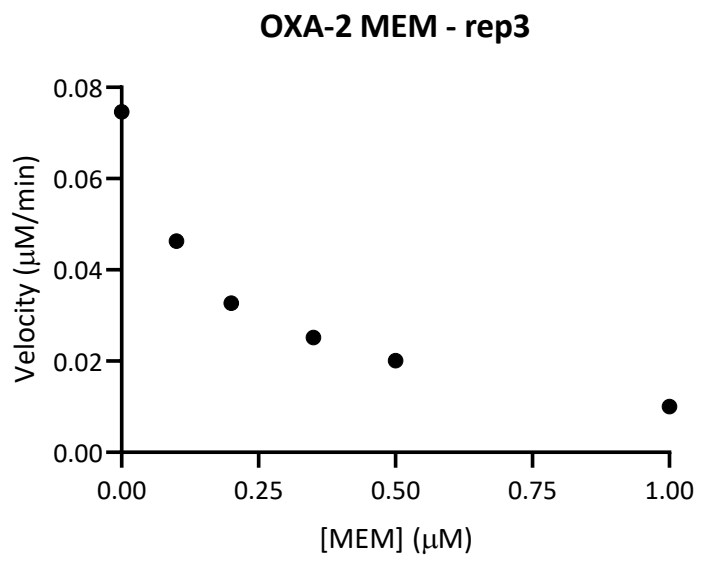	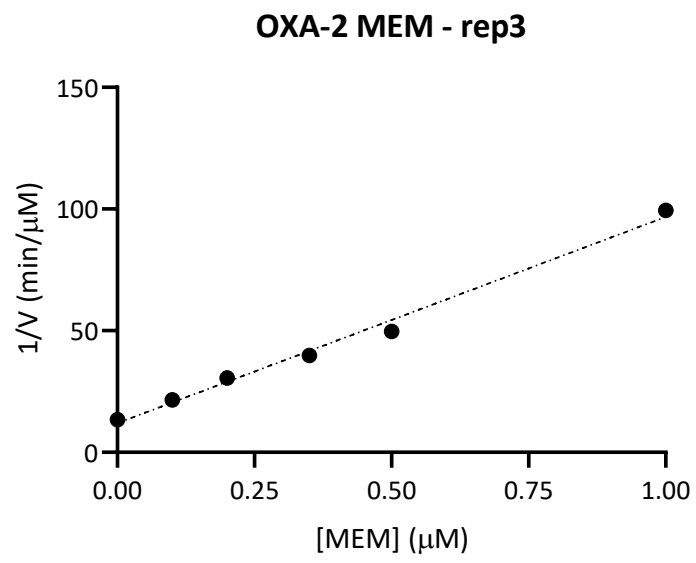	*y = 84.728x + 12.038*<br>*R² = 0.992*<br>$K_{i\ app\ (observed)}$ = 0.142 μM<br>$K_{i\ app\ (corrected)}$ = 0.021 μM

Table S5. Determination of maximum velocity (V_{max}) and k_{cat} values for ceftazidime, ceftolozane and meropenem hydrolysis by OXA-2 under saturating substrate concentrations. Individual values obtained from three independent experiments are shown.

Ceftazidime
High K_M values precluded the determination of V_{max} and, consequently, k_{cat} for OXA-2 and ceftazidime.

Ceftolozane						
	[S][a] (μM)	ΔAbs/min	V_{max} (μM/min)	V_{enzyme}[b] (μL)	$[E_t]$[c] (μM)	k_{cat} (s^{-1})
Rep 1	2000.000	-0.013	4894.763	10.000	37.291	0.219
Rep 2	2000.000	-0.009	3120.411	5.000	37.291	0.279
Rep 3	2000.000	-0.008	2936.858	5.000	37.291	0.263

Meropenem						
	[S][a] (μM)	ΔAbs/min	V_{max} (μM/min)	V_{enzyme}[b] (μL)	$[E_t]$[c] (μM)	k_{cat} (s^{-1})
Rep 1	300.000	-0.001	274.223	1.000	37.291	0.123
Rep 2	400.000	-0.001	191.956	1.000	37.291	0.086
Rep 3	400.000	-0.002	342.779	1.000	37.291	0.153

[a] Concentration of the β-lactam substrate used in the assay.

[b] Volume of enzyme solution added to the reaction mixture.

[c] Initial enzyme concentration in the stock solution.

Table S6. Michaelis-Menten curves and corresponding Lineweaver-Burk plots for nitrocefin (NCF) hydrolysis by OXA-1381. Results from three independent experiments are shown. For each experiment, the Michaelis-Menten curve and the corresponding Lineweaver-Burk linearization are presented together with the regression equation, coefficient of determination (R^2), and the calculated kinetic parameters (K_M, k_{cat} and k_{cat}/K_M).

Michaelis-Menten plots	Lineweaver-Burk	
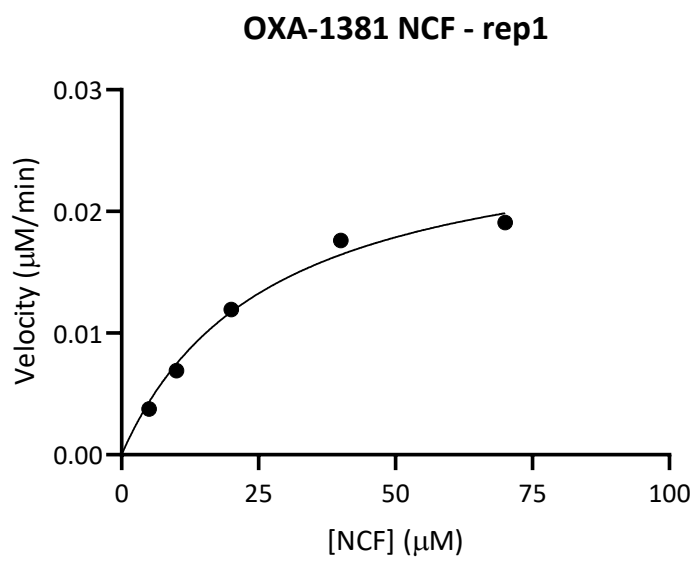	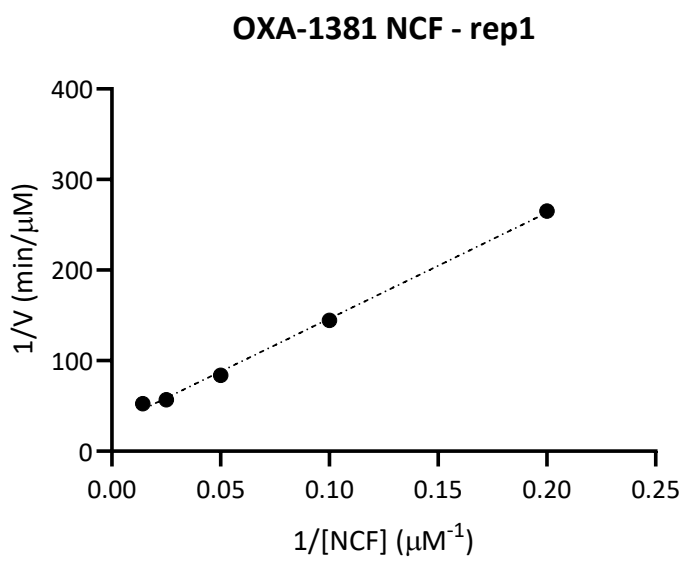	*y = 1168.681x + 29.496*<br>*R² = 0.998*<br><br>K_M = 39.621 μM<br>k_{cat} = 10.248 s^{-1}<br>k_{cat}/K_M = 258.644 mM^{-1} s^{-1}
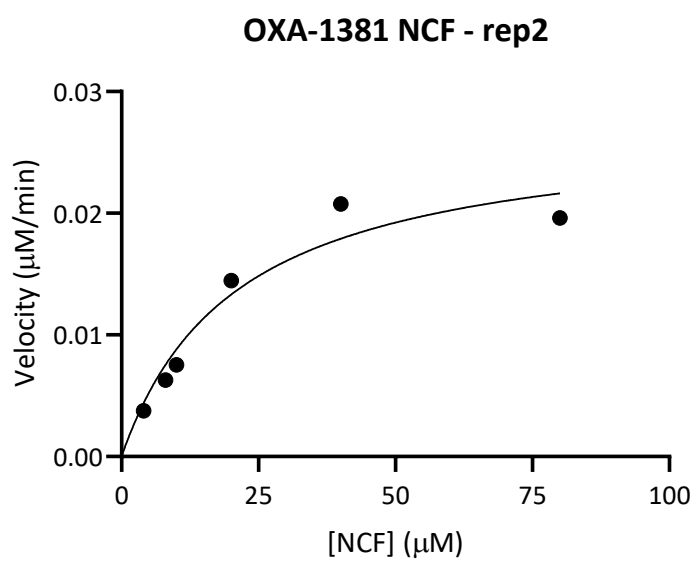	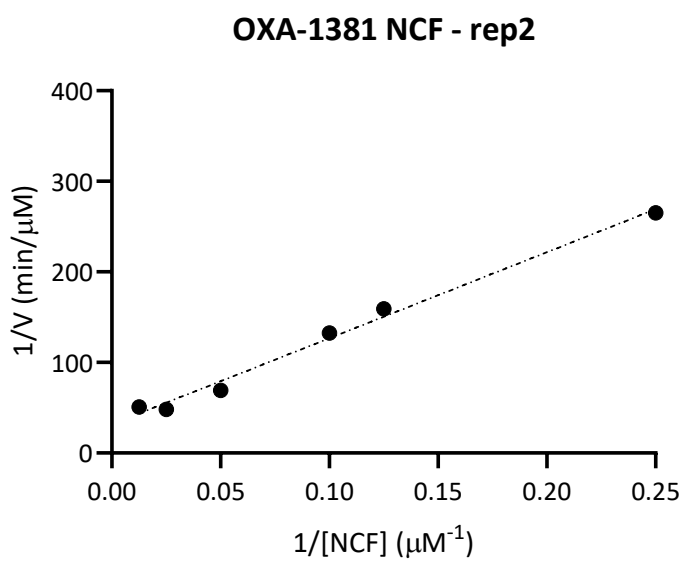	*y = 949.671x + 31.764*<br>*R² = 0.991*<br><br>K_M = 29.898 μM<br>k_{cat} = 9.516 s^{-1}<br>k_{cat}/K_M = 318.292 mM^{-1} s^{-1}
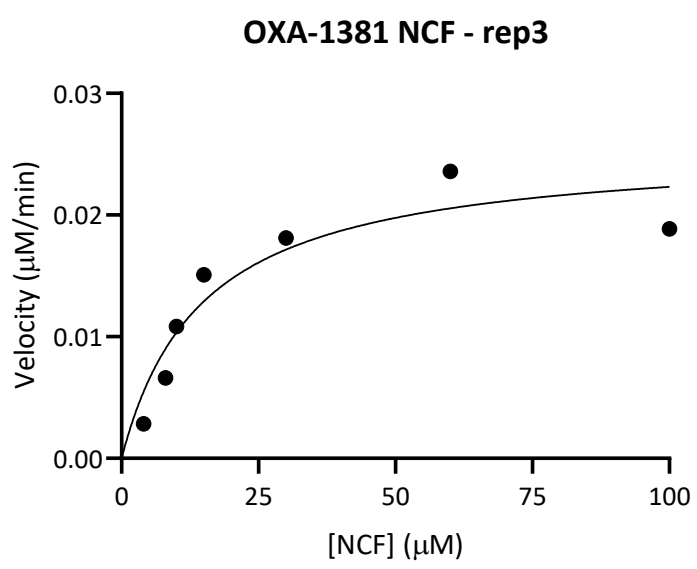	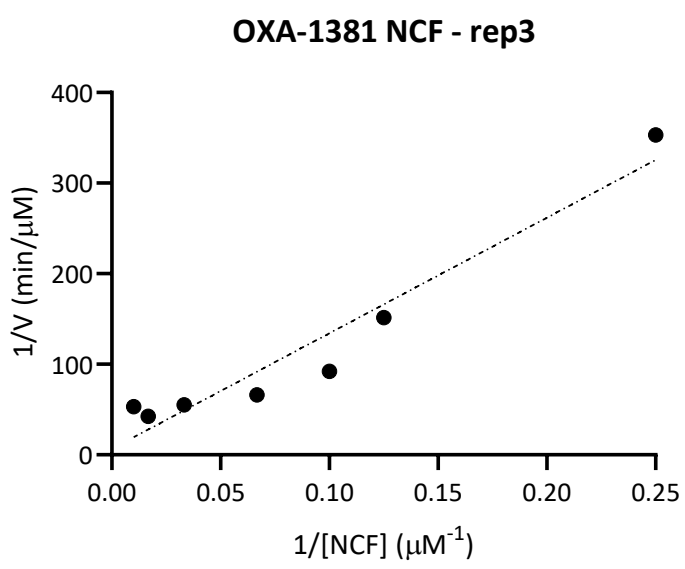	*y = 790.321x + 30.422*<br>*R² = 0.841*<br><br>K_M = 25.979 μM<br>k_{cat} = 9.936 s^{-1}<br>k_{cat}/K_M = 382.468 mM^{-1} s^{-1}

Table S7. Competitive inhibition assays for the determination of the apparent inhibition constant ($K_{i\ app}$) of ceftazidime (CAZ) against OXA-1381 using nitrocefin as the reporter substrate. Three independent experiments are shown. For each experiment, the linearization plot is presented together with the regression equation, coefficient of determination (R^2), and the calculated $K_{i\ app}$ values. Nitrocefin concentration was fixed at 150 µM.

**Nitrocefin vs Ceftazidime**	**Linearization**	
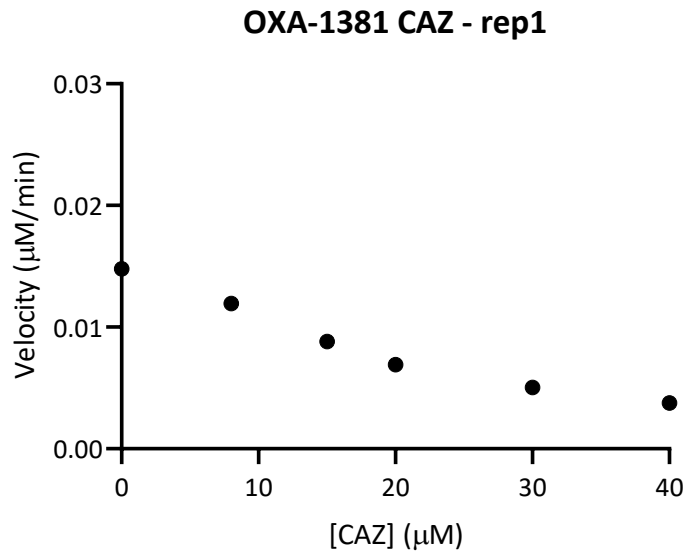	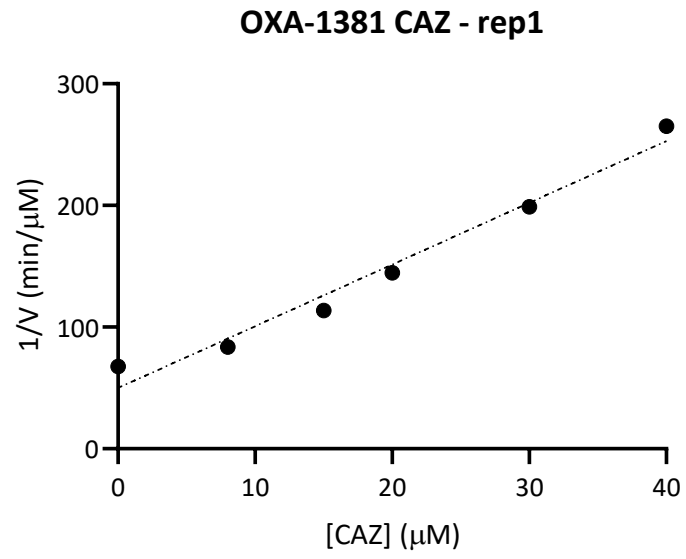	*$y = 5.072x + 50.005$*<br>*$R^2 = 0.974$*<br>$K_{i\ app\ (observed)}$ = 9.858 µM<br>$K_{i\ app\ (corrected)}$ = 1.726 µM
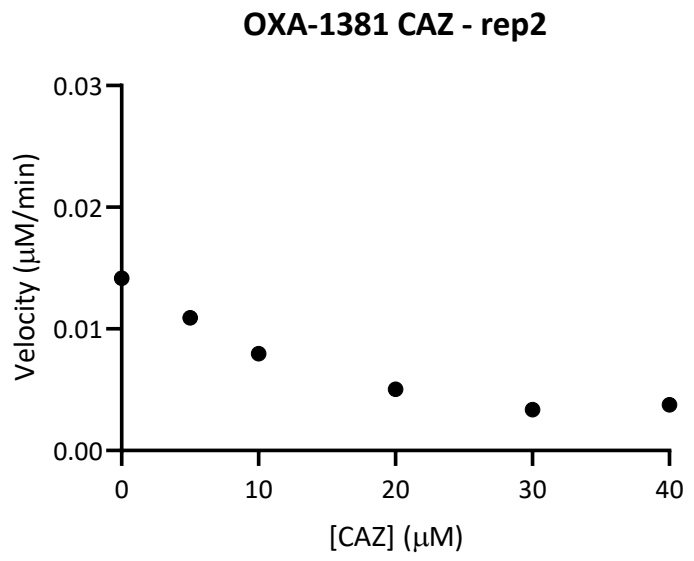	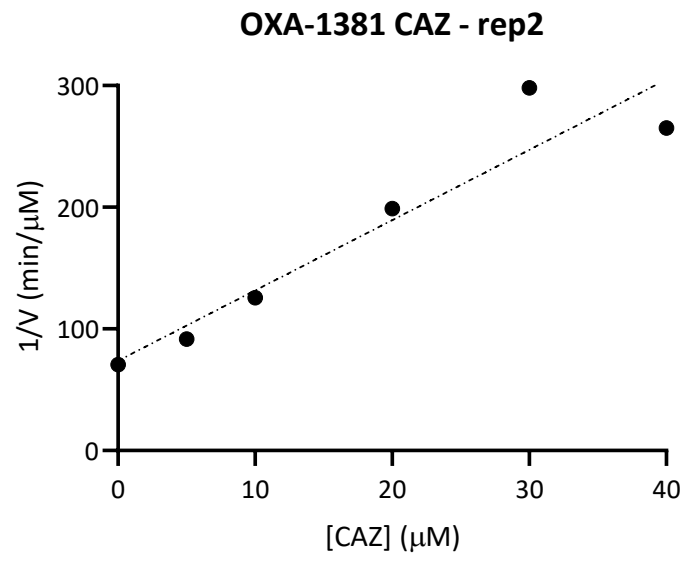	*$y = 5.778x + 73.854$*<br>*$R^2 = 0.899$*<br>$K_{i\ app\ (observed)}$ = 12.782 µM<br>$K_{i\ app\ (corrected)}$ = 2.238 µM
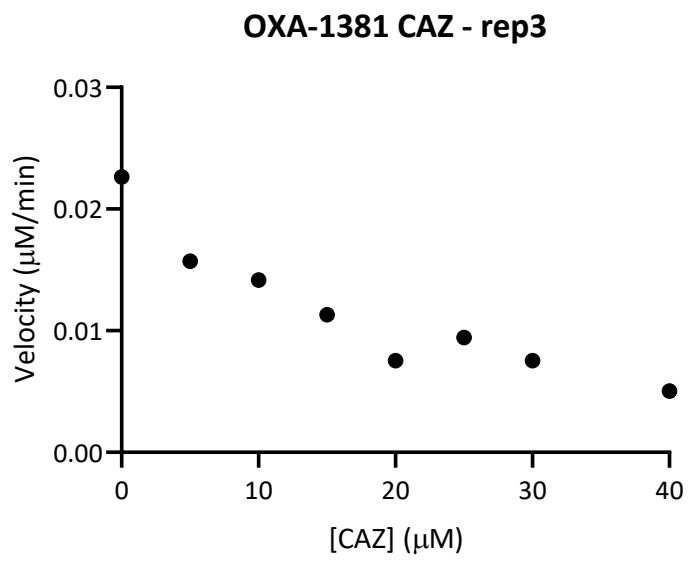	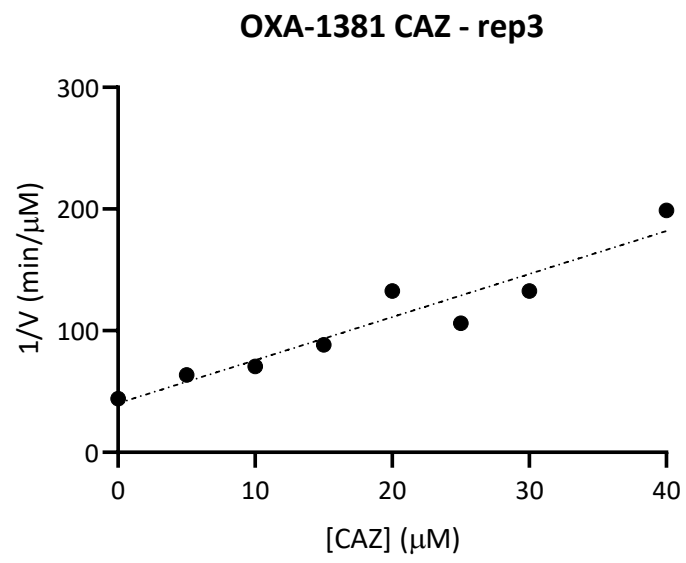	*$y = 3.5391x + 40.419$*<br>*$R^2 = 0.9094$*<br>$K_{i\ app\ (observed)}$ = 11.421 µM<br>$K_{i\ app\ (corrected)}$ = 1.999 µM

Table S8. Competitive inhibition assays for the determination of the apparent inhibition constant ($K_{i\ app}$) of ceftolozane (COZ) against OXA-1381 using nitrocefin as the reporter substrate. Three independent experiments are shown. For each experiment, the linearization plot is presented together with the regression equation, coefficient of determination (R^2), and the calculated $K_{i\ app}$ values. Nitrocefin concentration was fixed at 150 µM.

Nitrocefin vs Ceftolozane | **Linearization**

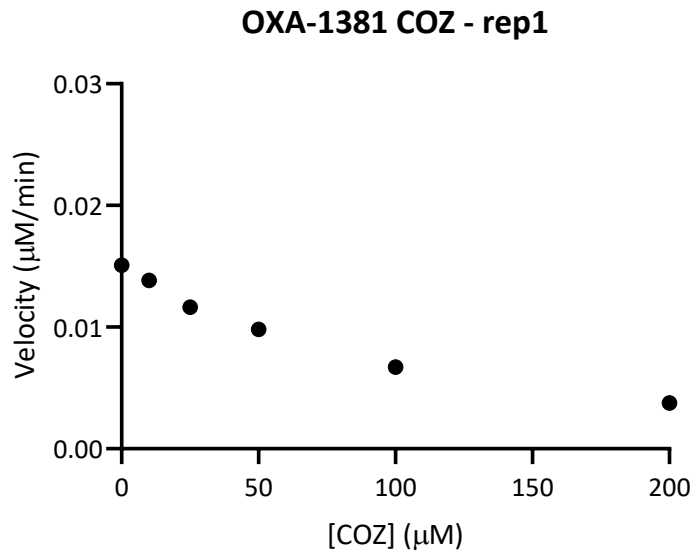

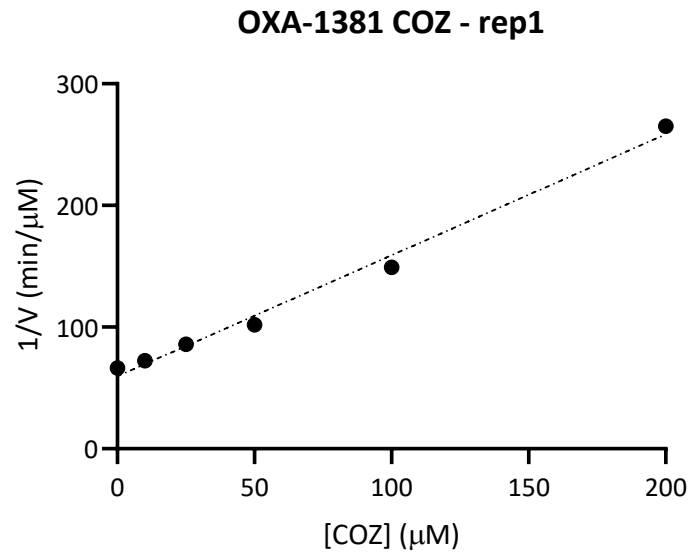

y = 0.994x + 59.601
R² = 0.991

$K_{i\ app\ (observed)}$ = 59.937 µM
$K_{i\ app\ (corrected)}$ = 10.492 µM

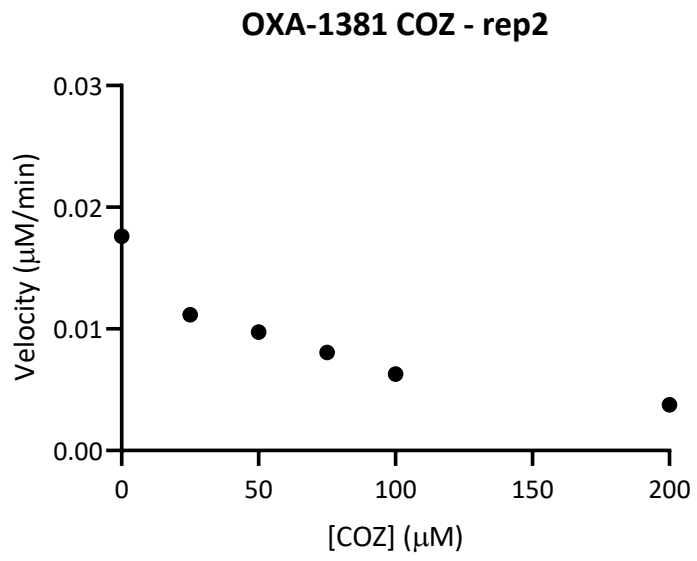

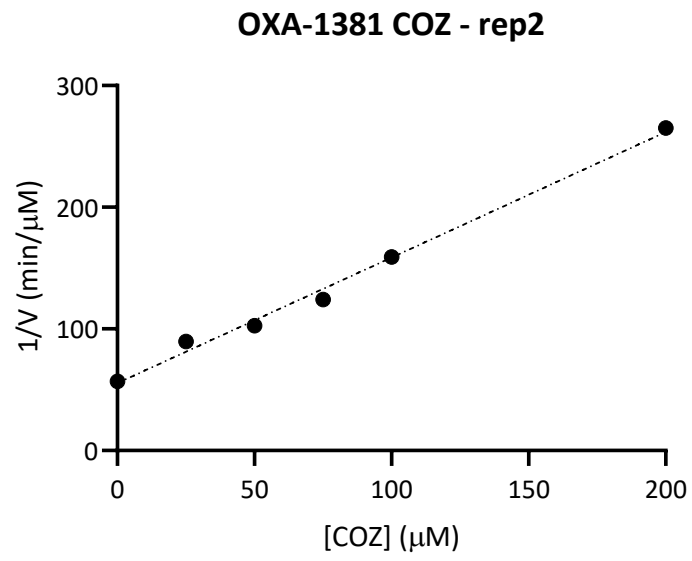

y = 1.032x + 55.467
R² = 0.993

$K_{i\ app\ (observed)}$ = 53.752 µM

$K_{i\ app\ (corrected)}$ = 9.410 µM

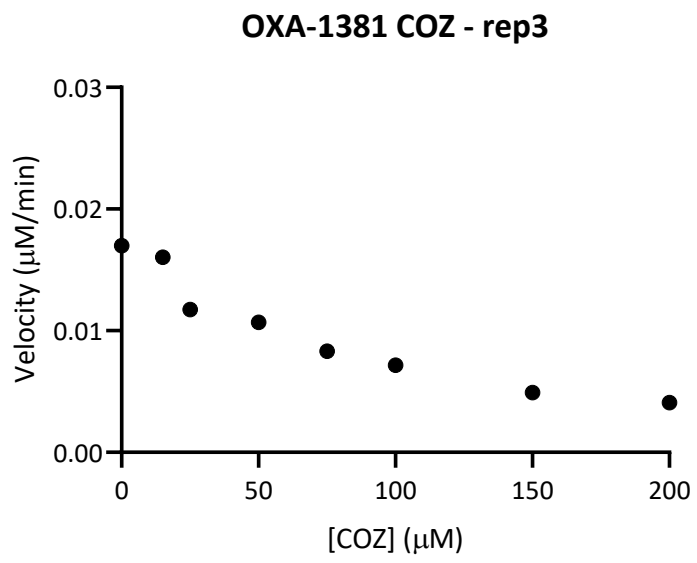

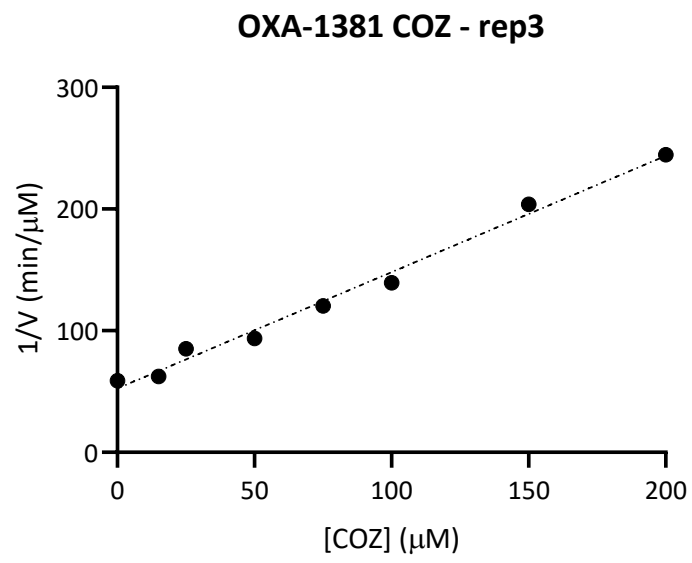

y = 0.956x + 52.518
R² = 0.989

$K_{i\ app\ (observed)}$ = 54.911 µM

$K_{i\ app\ (corrected)}$ = 9.612 µM

Table S9. Competitive inhibition assays for the determination of the apparent inhibition constant ($K_{i\ app}$) of meropenem (MEM) against OXA-1381 using nitrocefin as the reporter substrate. Three independent experiments are shown. For each experiment, the linearization plot is presented together with the regression equation, coefficient of determination (R^2), and the calculated $K_{i\ app}$ values. Nitrocefin concentration was fixed at 150 µM.

Nitrocefin vs Meropenem	Linearization	

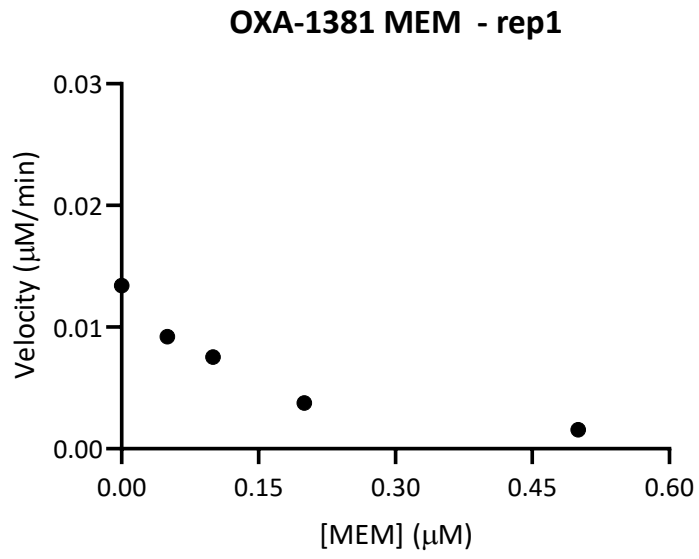

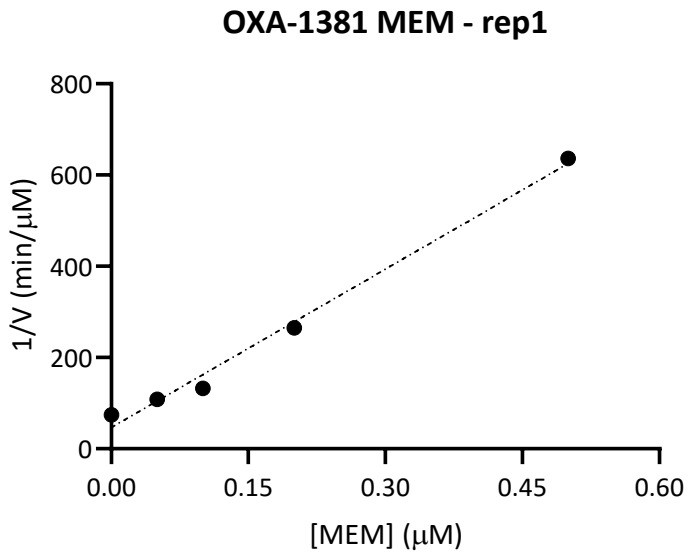

y = 1157.440x + 46.523
R^2 = 0.991

$K_{i\ app\ (observed)}$ = 0.040 µM

$K_{i\ app\ (corrected)}$ = 0.007 µM

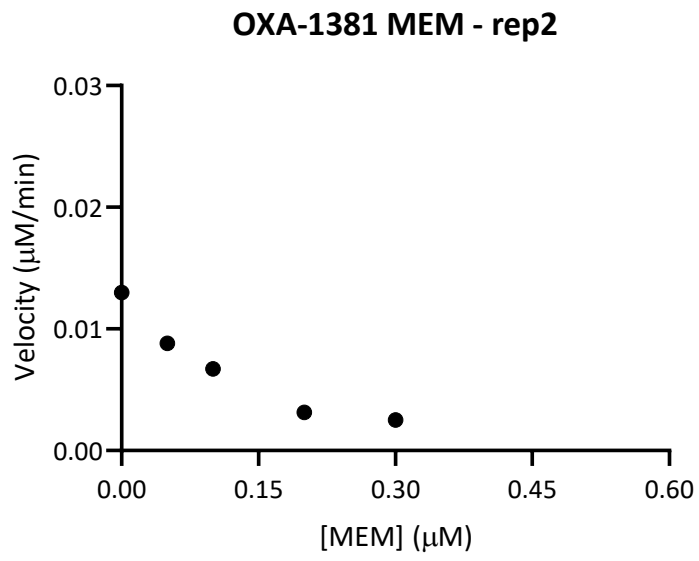

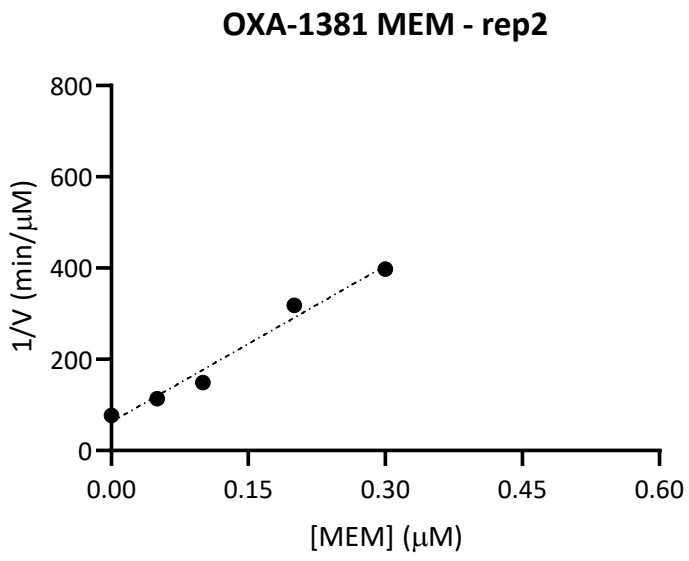

y = 1142.686x + 62.465
R^2 = 0.977

$K_{i\ app\ (observed)}$ = 0.055 µM

$K_{i\ app\ (corrected)}$ = 0.010 µM

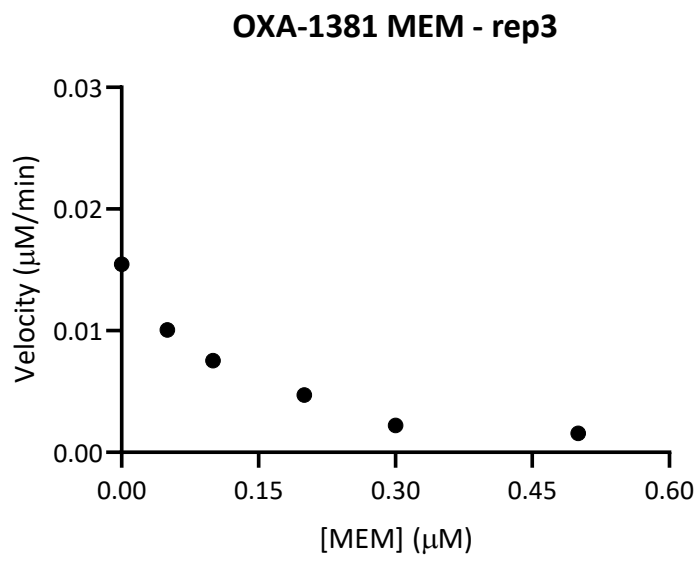

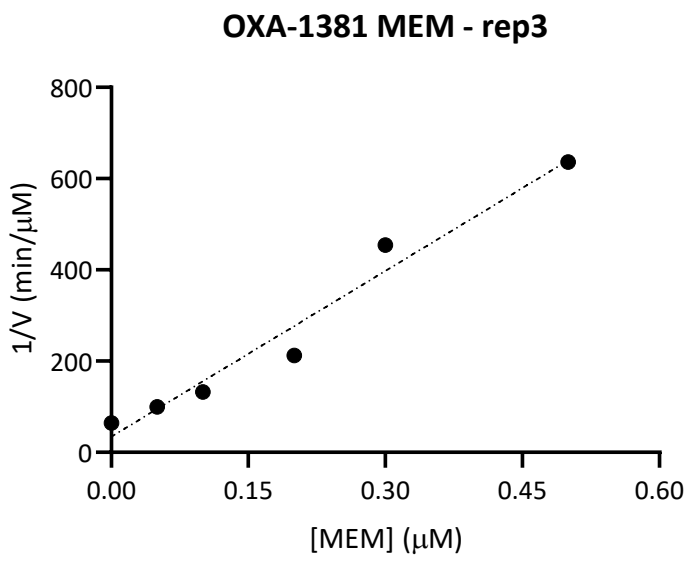

y = 1149.688x + 65.446
R^2 = 989

$K_{i\ app\ (observed)}$ = 0.057 µM

$K_{i\ app\ (corrected)}$ = 0.010 µM

Table S10. Determination of maximum velocity (V_{max}) and k_{cat} values for ceftazidime, ceftolozane and meropenem hydrolysis by OXA-1381 under saturating substrate concentrations. Individual values obtained from three independent experiments are shown.

Ceftazidime						
	[S][a] (µM)	ΔAbs/min	V_{max} (µM/min)	V_{enzyme}[b] (µL)	$[E_t]$[c] (µM)	k_{cat} (s^{-1})
Rep 1	100.000	-0.008	2370.203	5.000	55.138	0.143
Rep 2	500.000	-0.001	406.321	1.000	55.138	0.123
Rep 3	500.000	-0.001	395.034	1.000	55.138	0.119

Ceftolozane						
	[S][a] (µM)	ΔAbs/min	V_{max} (µM/min)	V_{enzyme}[b] (µL)	$[E_t]$[c] (µM)	k_{cat} (s^{-1})
Rep 1	50.000	-0.007	2517.306	1.000	55.138	0.761
Rep 2	75.000	-0.007	2466.960	1.000	55.138	0.746
Rep 3	100.000	-0.005	1908.957	1.000	55.138	0.577

Meropenem						
	[S][a] (µM)	ΔAbs/min	V_{max} (µM/min)	V_{enzyme}[b] (µL)	$[E_t]$[c] (µM)	k_{cat} (s^{-1})
Rep 1	200.000	-0.001	274.223	5.000	55.138	0.017
Rep 2	350.000	-0.001	182.815	5.000	55.138	0.011
Rep 3	500.000	-0.002	446.179	10.000	55.138	0.014

[a] Concentration of the β-lactam substrate used in the assay.

[b] Volume of enzyme solution added to the reaction mixture.

[c] Initial enzyme concentration in the stock solution.

Table S11. Linear regression plots of the inverse of the initial velocity (1/V) versus avibactam (AVI) concentration used to calculate the IC_{50} of avibactam for OXA-2. Three independent experiments are shown together with the corresponding regression equations, coefficients of determination (R^2), and calculated IC_{50} values.

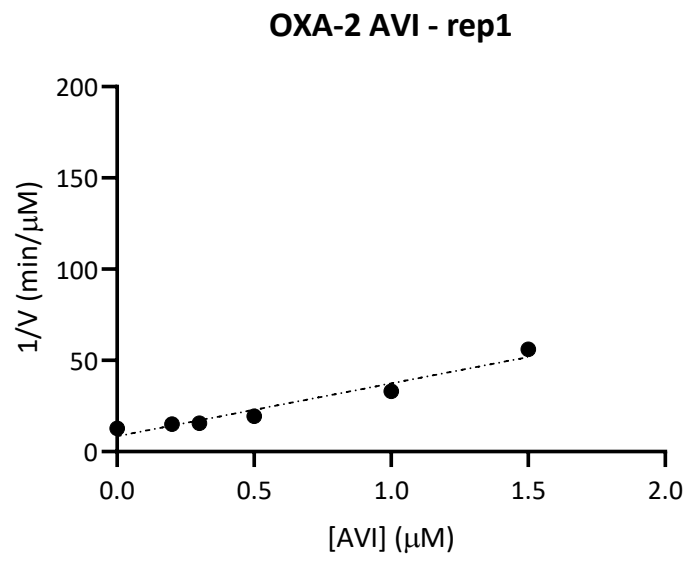

y = 28.928x + 8.463
$R^2 = 0.951$

IC_{50} = 0.590 μM

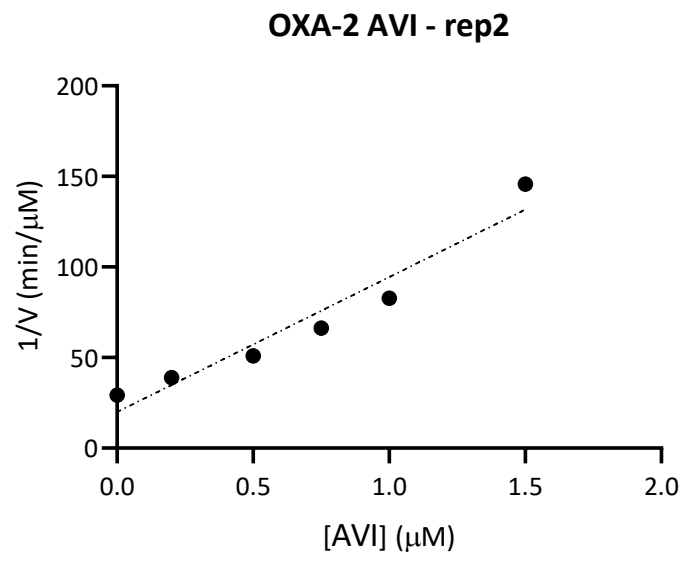

y = 74.475x + 19.966
$R^2 = 0.936$

IC_{50} = 0.517 μM

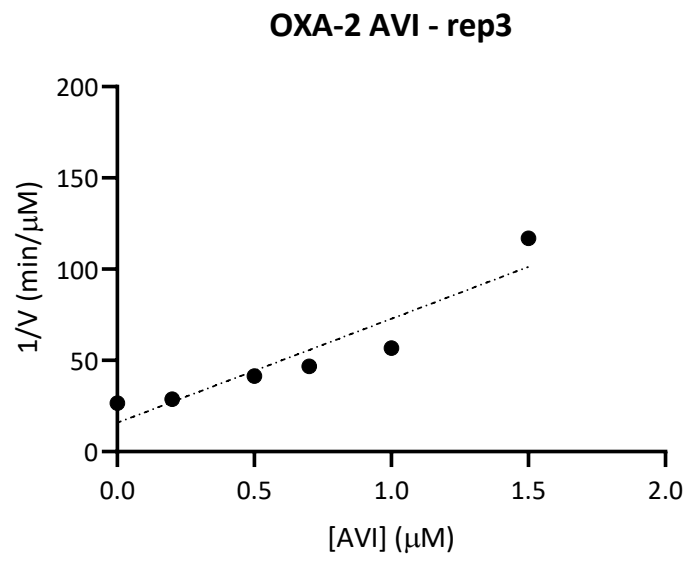

y = 56.984x + 15.823
$R^2 = 0.873$

IC_{50} = 0.652 μM

Table S12. Linear regression plots of the inverse of the initial velocity (1/V) versus tazobactam (TAZ) concentration used to calculate the IC_{50} of tazobactam for OXA-2. Three independent experiments are shown together with the corresponding regression equations, coefficients of determination (R^2), and calculated IC_{50} values.

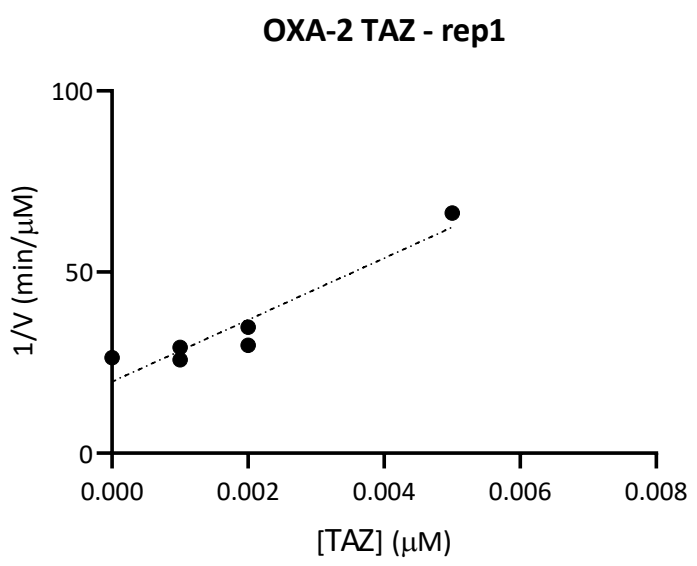

y = 8464.078x + 21.256
$R^2 = 0.948$

IC_{50} = 0.004 μM

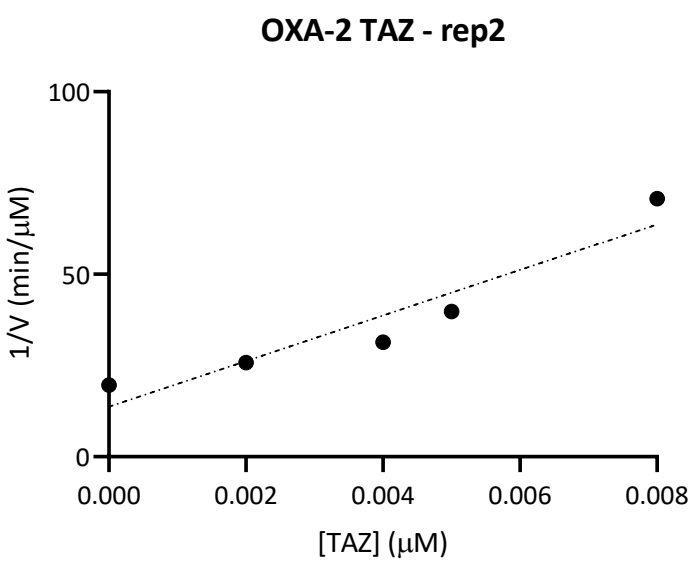

y = 6609.354x + 13.650
$R^2 = 0.894$

IC_{50} = 0.004 μM

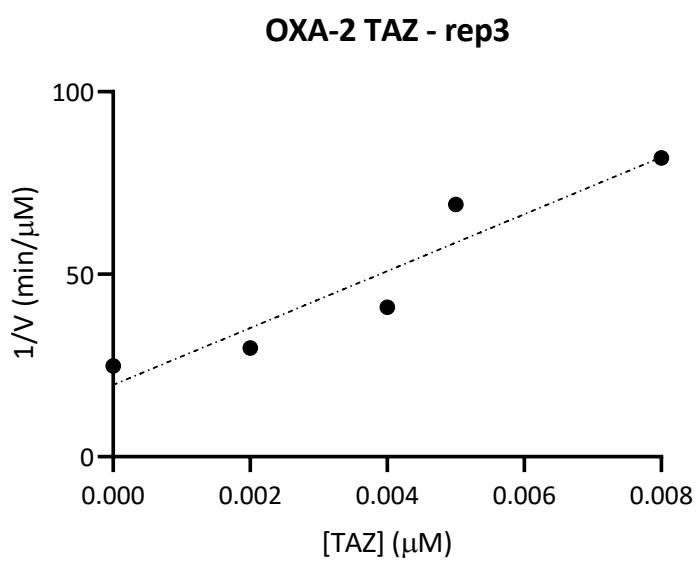

y = 12511.518x + 8.468
$R^2 = 0.892$

IC_{50} = 0.003 μM

Table S13. Linear regression plots of the inverse of the initial velocity (1/V) versus avibactam (AVI) concentration used to calculate the IC_{50} of avibactam for OXA-1381. Three independent experiments are shown together with the corresponding regression equations, coefficients of determination (R^2), and calculated IC_{50} values.

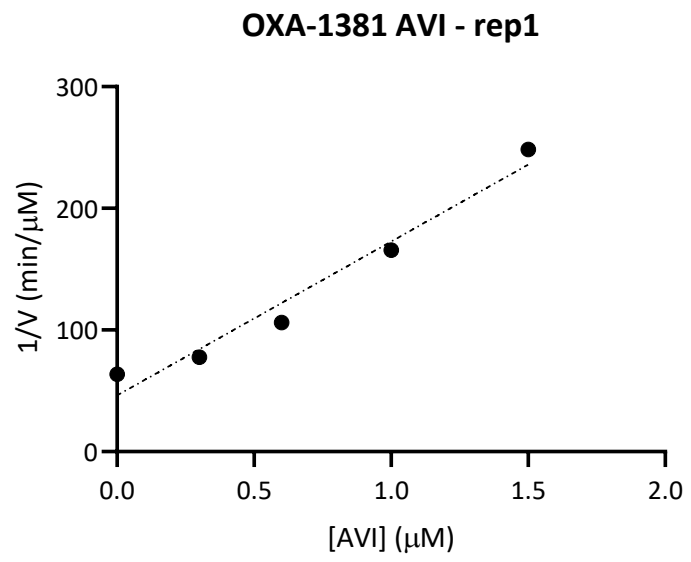

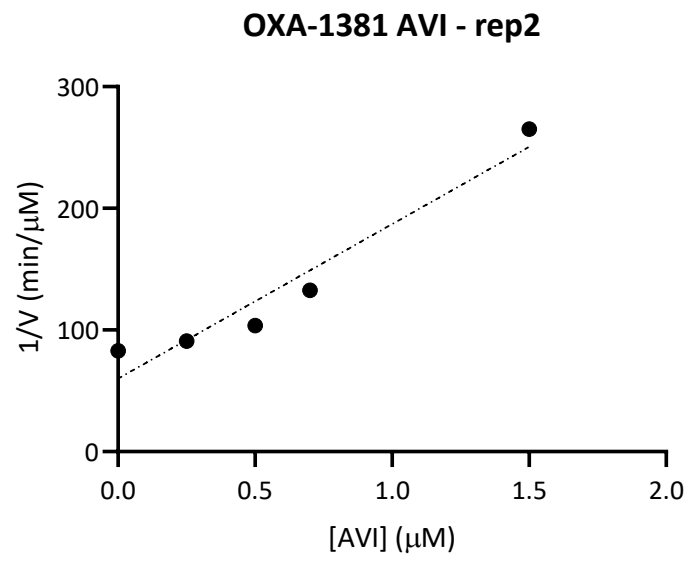

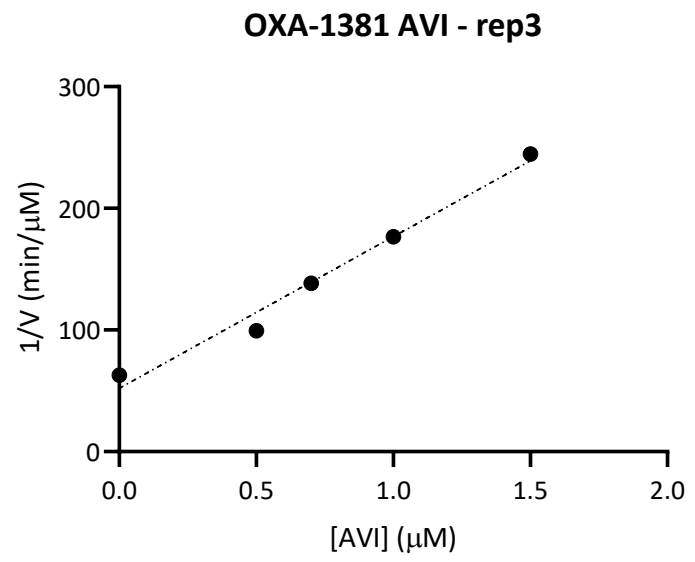

rep1	rep2	rep3
y = 164.540x + 28.366	*y = 160.037 + 46.697*	*y = 124.613x + 52.122*
$R^2 = 0.942$	$R^2 = 0.951$	$R^2 = 0.981$
IC_{50} = 0.600 µM	IC_{50} = 0.743 µM	IC_{50} = 0.589 µM

Table S14. Linear regression plots of the inverse of the initial velocity (1/V) versus tazobactam (TAZ) concentration used to calculate the IC_{50} of tazobactam for OXA-1381. Three independent experiments are shown together with the corresponding regression equations, coefficients of determination (R^2), and calculated IC_{50} values.

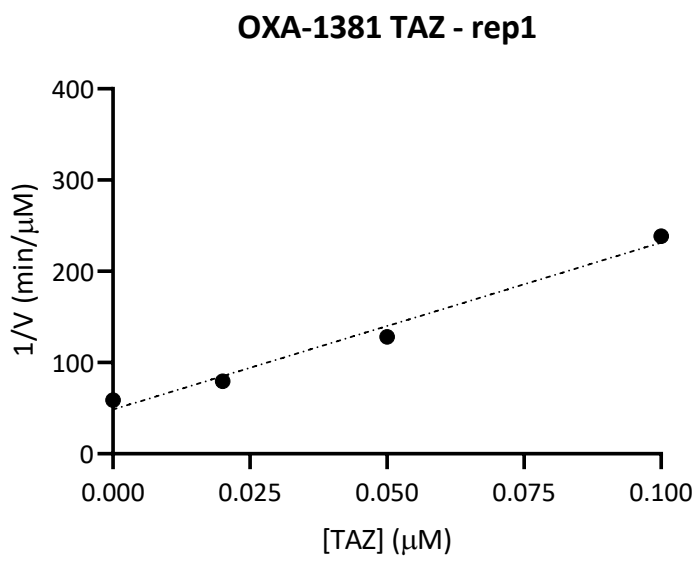

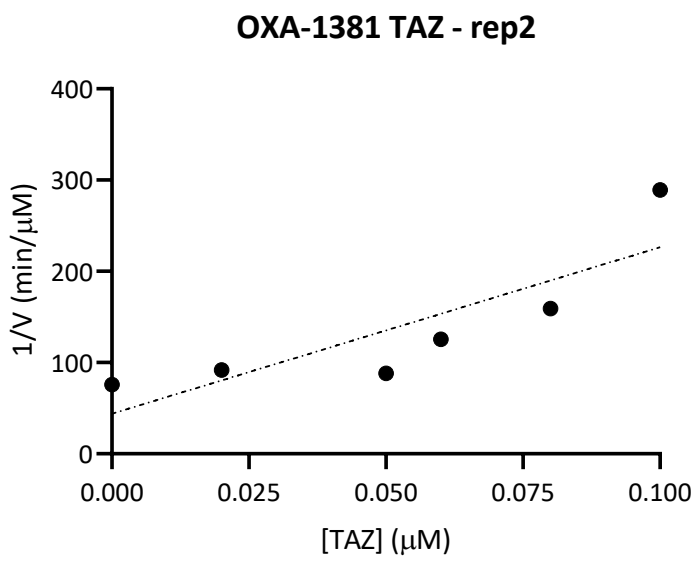

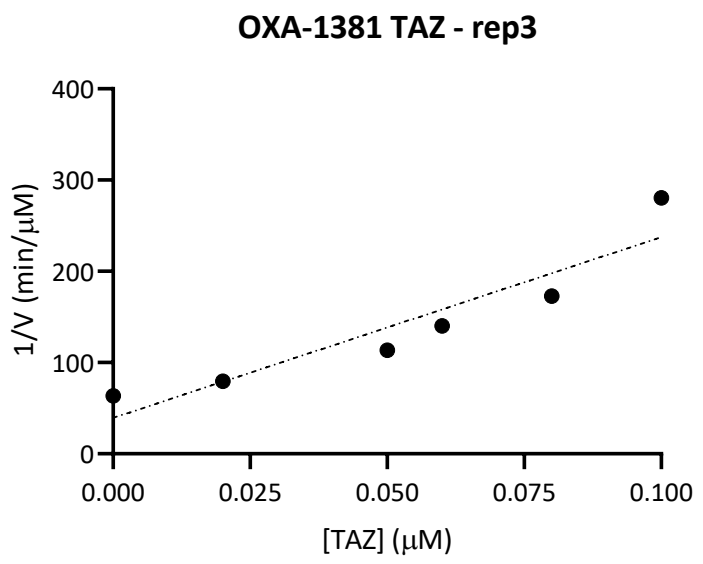

rep1	rep2	rep3
y = 1829.765x + 48.514	*y = 1824.675x + 43.958*	*y = 1367.906x + 56.506*
$R^2 = 0.983$	$R^2 = 0.718$	$R^2 = 0.969$
IC_{50} = 0.038 µM	IC_{50} = 0.059 µM	IC_{50} = 0.052 µM
